# The Diversity-Weighted Living Planet Index: Controlling for Taxonomic Bias in a Global Biodiversity Indicator

**DOI:** 10.1371/journal.pone.0169156

**Published:** 2017-01-03

**Authors:** Louise McRae, Stefanie Deinet, Robin Freeman

**Affiliations:** Indicators and Assessments Research Unit, Institute of Zoology, Zoological Society of London, United Kingdom; University of Hyogo, JAPAN

## Abstract

As threats to species continue to increase, precise and unbiased measures of the impact these pressures are having on global biodiversity are urgently needed. Some existing indicators of the status and trends of biodiversity largely rely on publicly available data from the scientific and grey literature, and are therefore prone to biases introduced through over-representation of well-studied groups and regions in monitoring schemes. This can give misleading estimates of biodiversity trends. Here, we report on an approach to tackle taxonomic and geographic bias in one such indicator (Living Planet Index) by accounting for the estimated number of species within biogeographical realms, and the relative diversity of species within them. Based on a proportionally weighted index, we estimate a global population decline in vertebrate species between 1970 and 2012 of 58% rather than 20% from an index with no proportional weighting. From this data set, comprising 14,152 populations of 3,706 species from 3,095 data sources, we also find that freshwater populations have declined by 81%, marine populations by 36%, and terrestrial populations by 38% when using proportional weighting (compared to trends of -46%, +12% and +15% respectively). These results not only show starker declines than previously estimated, but suggests that those species for which there is poorer data coverage may be declining more rapidly.

## Introduction

Accurately quantifying trends in global biodiversity is crucial in order to understand the impacts of threats on the species and ecosystems on which humans rely [1]. The need for such metrics is pressing as threats and pressures upon the natural world continue largely unabated [2,3] and recent estimates of species extinction rates suggest they are significantly higher than background rates, having risen dramatically over the last 200 years [4,5]. Strategic Goal C of the Aichi Biodiversity Targets [6] aims ‘to improve the status of biodiversity by safeguarding ecosystems, species and genetic diversity’. In particular, Aichi Target 12 focusses on preventing the extinction of threatened species and improving and sustaining their conservation status. The mechanism required to assess progress towards this target relies on the development of robust and quantitative measures of the status of and trends in biodiversity and in this case, a focus on species [3].

The Living Planet Index (LPI) [7–9], one in the suite of global species indicators used to track progress towards Aichi Target 12, focusses on monitoring the population trends of vertebrate species. The LPI includes available published data, primarily in the scientific and grey literature (e.g. government/NGO reports) taken from the Living Planet Database (LPD) and records trends in 14,152 populations of 3,706 species. However, its reliance on available data means there is bias in the LPD resulting from the taxonomic and geographical distribution of the data used [8]. These types of bias are a common feature of other global biodiversity databases [10,11], usually with a noticeable gap in data from tropical regions [12]. The disparity in spatial coverage particularly reiterates that, in a time of persistent biodiversity decline, there are many gaps in our knowledge of the exact patterns and extent of this global problem [13]. Furthermore, the performance of biodiversity indicators such as the LPI can be compromised by the presence of bias in the data and limited in effectiveness as tools in measuring progress towards specific policy targets [1,14].

Other indicators based on species abundance (e.g. [15,16]) are developed for a selected group of species using a systematic monitoring protocol to collect the data used, so the indicator is spatially and taxonomically representative of the region and taxa in question. However, no indicator of this kind yet exists which has a global extent and covers taxonomic groups beyond birds and butterflies [15,16]. There is a tradeoff to be made between the time and resources required to develop a representative global monitoring scheme and the need to measure and report on biodiversity change [1]. In light of this, it can be prudent and cost-effective in the near term to build on existing indicators provided there is an understanding of any effects from the bias that they contain [17].

The database behind the Living Planet Index has been continually augmented since its inception in 1998 [18] and data are still being added (S1 Fig). In light of the applicability of the Living Planet Index as a global biodiversity indicator [3] and given the ongoing need for reporting tools for current and new targets for biodiversity, such as the Aichi Targets [6] and Sustainable Development Goals [19], we aim to continue the development of the LPI by both filling data gaps and by addressing the existing bias in the indicator. Here, we describe an approach which tackles the latter. We collated estimates of the known number of species across biogeographical realms and assessed the representativeness of the Living Planet Index database for species groups within these. We then developed the diversity weighted Living Planet Index which attempts to make the estimated index more representative of vertebrate biodiversity by accounting for the estimated diversity of species.

## Materials and Methods

### Data collection for the LPI

All data used in constructing the LPI are time series of either population size, density, abundance or a proxy of abundance. The species population data used to calculate the index are gathered from a variety of sources. Time series information for vertebrate species is collated from published scientific literature, online databases and grey literature (government/NGO reports), totaling 3,095 individual data sources. Data are only included if a measure of population size is available for at least two years, and information available on how the data were collected, what the units of measurement were, and the geographic location of the population. The data must be collected using the same method on the same population throughout the time series and the data source referenced and traceable (see [8] for further details).

The period covered by the index is from 1970 to 2012. The year 2012 is chosen as the cut-off point for the index because at present there are insufficient data to calculate a robust index after this point due to publication time-lag. Data sets are continually being added to the database. In addition to the population data, each time series is assigned to a system–terrestrial, freshwater and marine–based on both the location of the monitored population and the habitat the species mostly relies on. The geographic coordinates of the location are used to assign each population time series to a land-based or marine biogeographic realm (S2 Fig).

We examined the pattern of geographic bias in a data set which relies on using published data, in two ways. The first was to create a display of the broad spatial pattern of the LPD by mapping the location of each population time series onto a map depicting global vertebrate species richness (reproduced from [20]). Secondly, we followed the approach taken by Martin, et al [21] to analyse the geographic bias among terrestrial ecological study sites. Using the unique locations in the terrestrial component of the LPD we calculated the proportion of sites that are protected, the proportion in different woodland biomes and the proportion that occur in wealthy countries (S1 Appendix). We then compared this to the findings from Martin et al.

### Assessing species representation

Numbers of species in the LPI database were compared with estimates of the number of known species in each of the following subcategories: system (terrestrial, freshwater, marine); taxonomic group (birds, mammals, reptiles, amphibians, fishes); land-based biogeographic realm for terrestrial and freshwater species (Afrotropical, Australasia, Indo-Malaya, Nearctic, Neotropical, Oceania, Palearctic); marine realm for marine species (Arctic, Atlantic north temperate, Atlantic tropical and subtropical, Pacific north temperate, Tropical and subtropical Indo-Pacific, Southern temperate and Antarctic).

Terrestrial and freshwater bird, mammal, reptile and amphibian species numbers were obtained from the WWF Wildfinder database [22]. This database lists extant species within each ecoregion. From this database, we extracted species lists and totals for the terrestrial and freshwater biogeographic realms. Freshwater fish species numbers were extracted from the Freshwater Ecoregions of the World data set [23] which also had ecoregion level species lists which we amalgamated into biogeographic realm lists.

Bird, mammal, reptiles and amphibian species numbers were further split into terrestrial and freshwater groups according to the habitat information on their species account on the IUCN Red List 2016.2 [24]. Species which were categorized as exclusively terrestrial or freshwater were placed in the relevant list. Species which were listed as both terrestrial and freshwater were placed in both, so these system lists are not mutually exclusive which mirrors the LPI database where species can be assigned to both terrestrial and freshwater systems.

In some cases, taxonomic discrepancies meant that it was not clear whether a species should be categorized as freshwater or terrestrial. To minimize this, we conducted synonym searches in the Red List taxonomic fields to increase matches and identify unique orders, families or genera that should be classified as exclusively terrestrial or freshwater. Any remaining species that were not matched were kept in both terrestrial and freshwater lists. For reptile species not assessed by the IUCN Red List, we based the decision on the system assigned to other species of the same genera or family level. Alternatively we searched for habitat preferences for the species on the Reptile Database [25].

Marine fish, bird and reptile species totals were obtained by searching for ‘Pisces’, ‘Aves', and ‘Reptilia’ respectively within a polygon drawn for each marine realm from the Ocean Biogeographic Information System [26]. Species totals for marine mammals were obtained through advanced searches on the IUCN Red List to identify total numbers of marine mammals occurring in each FAO marine area [24]. The FAO marine areas were then assigned to the appropriate marine realm in order to estimate total species number for each realm.

For each realm, we then compared the estimated proportion of species from each taxonomic group within each realm with the proportions of species found in the LPI for that realm. We did this for terrestrial, freshwater and marine species separately. Binomial tests were used to assess significant over or under-representation. We assessed the impact of removing low representation (less than 1%) on the resulting indices. We also investigated whether the proportion of species in the LPI database assessed as threatened on the IUCN Red List [24] differed significantly from the actual proportions of threatened species within five of the extinction risk categories (Least Concern, Near Threatened, Vulnerable, Endangered, Critically Endangered) and for each taxonomic group on the IUCN Red List. We did not compare proportions in the Data Deficient, Extinct or Extinct in the Wild categories as we would not anticipate having population trends data for such species in the LPD. For reptiles and fishes which have not been comprehensively assessed, we used estimates of proportion threatened from those species that have been assessed. As an extension of this analysis, we replicated the comparison removing any threatened species that had not been assessed under Criterion A, which is based on a reduction in population size. Species assessed under other criteria might not necessarily show population declines, so this approach aims to test for a bias towards threatened species that do have declining populations.

### Calculating the LPI

To facilitate easy replication of the results presented here, an r package, *rlpi*, for calculating the Living Planet Index using either approach outlined below is provided with tutorial documentation, example data sets and the publically available records from the Living Planet Database [27] at https://github.com/Zoological-Society-of-London/rlpi. The Living Planet Database contains a number of abundance records that have been provided in confidence. These are used to calculate the presented trends and statistics, but cannot be made publically available. We calculated the geometric mean of trends for each species within a Generalised Additive Modelling (GAM) framework, following [8], whereby each population time series with six or more data points was modelled using a GAM. Population time series with fewer than six data points or that resulted in poor GAM fit were modelled using the chain method [9]. Where we had more than one population time series for a species, the modelled annual trends *d*_*t*_ for each population were averaged to provide a single set of annual trends for each species:

$${\bar{d}}_{t}=\frac{1}{n_{t}}\sum_{i=1}^{n_{t}}d_{it}$$ (1)

where *n*_*t*_ is the number of populations, *d*_*t*_ is the annual rate of change for a population in a given year, given by

$$d_{t}=\log_{10}(\frac{N_{t}}{N_{t-1}})$$ (2)

where *N* is the population measure and *t* is the year.

Having constructed species, group, regional or global trends, these can be converted back to index values by:

$$I_{t}=I_{t-1}*10^{{\bar{d}}_{t}},\;I_{0}=1$$ (3)

Throughout the following processes, we refer to ‘averaging’ trends–in all cases, we refer to averaging lambda values, prior to converting them to index values–generating the geometric mean abundance. This final step only occurs after all other steps have taken place.

We used two approaches for calculating a global scale index. The first, unweighted method (LPI-U), follows the process outlined in [8] whereby the data are divided into six subsets based on region (tropical or temperate) and the three systems (terrestrial, freshwater & marine) within each region. Indices for each system (tropical terrestrial, temperate freshwater, etc.) are calculated by averaging species trends within them. Separate tropical and temperate indices are then calculated by averaging the trends for each system. The tropical and temperate indices are finally averaged to produce a global scale LPI. This process of hierarchical averaging addresses some of the geographical disparity in the data set by equally weighting tropical and temperate regions but does not address taxonomic disparity or apply any proportional weighting.

**Fig 1:**
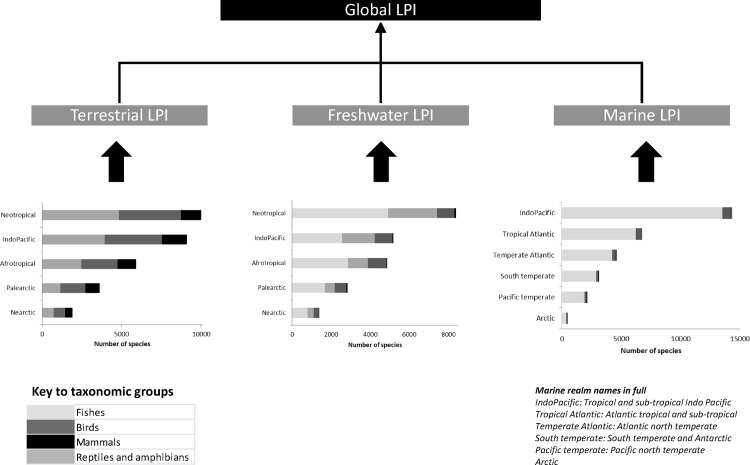
Schematic of the weighting process. Systems (Terrestrial/Freshwater/Marine) are weighted equally. Within each system, the proportion of species found across the realms that compose that system (the length of the bars above) is used to proportionally weight each realm's index. Within each realm, the diversity of species is used to weight taxonomic indices (the size of the grey-scale sections of the bars above).

The second approach, the diversity weighted LPI (LPI-D), incorporates a proportionally weighted system based on the species richness estimates described above (building upon suggestions in [8,9]). Because the reptile and amphibian data sets are small, these were combined into one herpetological group (‘herps’), leaving four species groups ('Birds', 'Mammals', 'Fish' and 'Herps'). For the same reason, we joined the biogeographic realms Australasia, Oceania and Indo-Malaya into one combined realm (‘Indo-Pacific’). The final data set comprised 57 subsets which incorporated each system, realm and taxonomic group combination (Fig 1).

Within each system and realm combination, the average species trend for each taxonomic group was then given a proportional weight according to estimated species richness (S10 Table, S11 Table). For example, birds represent 43.3% of terrestrial vertebrate species in the Palearctic so this value is used in the weighted average to construct the Palearctic realm trend for terrestrial species. This method of a weighted average was used to produce 16 trends for each system/realm combination. Summary pseudocode for this process is presented in Box 1. For example, in calculating the trends for freshwater Afro-tropical species, we weight taxonomic groups using their calculated proportions:

$${\bar{d}}_{t,\;FW\_AT}=\frac{1}{N_{T}}\sum_{j=1}^{N_{T}}{\bar{d}}_{jt}.w_{j}$$ (4)

where *N*_*T*_ is the number of taxonomic groups within the realm in question, *W*_*j*_ is the estimated proportion of species that that group represents (S10 Table, S11 Table), and *d*_*jt*_ is the calculated average trend in abundance for that taxonomic group at time *t*.

**Box 1. Pseudocode outlining the algorithm for constructing the global Living Planet Index.**

**For each species,** estimate rates of change:

    **For each population,**

      Estimate population lambdas (rates of change):

    Average population lambdas for each species to obtain species trend

**For each System** (terrestrial, freshwater, marine):

    **For each biogeographical realm** (Palearctic, Indo-Pacific, etc):

      **For each taxonomic group** (birds, mammals, fish, herps):

        Average species trends within group

      Average taxonomic trends, using taxonomic weightings, obtaining realm trend

    Average biogeographical realm trends, using realm weightings, obtaining system trend

Average system trends equally.

Convert average system rates of change to index values

The next stage was to produce three system-level trends (terrestrial, freshwater and marine). Each realm trend for that system was given a weighted value according to the proportion of species that the realm represents derived from the estimated number of known species. For example Palearctic species account for 10.6% of known terrestrial vertebrate species, so this value is used to weight the terrestrial Palearctic trend within the terrestrial index. This method of weighting was used to produce three indices for terrestrial, freshwater and marine which are then averaged to produce a single global trend as in [8]. This trend is indexed with the baseline of 1970 set to a value of 1.

As a smaller scale illustrative example, we calculated an index for the Palearctic realm using the two approaches described above. For the LPI-U approach, an average was taken of all terrestrial and freshwater species trends to produce the realm index. For the LPI-D approach, the index was calculated using a weighted average based on the combined proportion of terrestrial and freshwater species estimated for the Palearctic (see S10 Table, Palearctic column).

For each index, we generated 95% confidence intervals using a bootstrap resampling technique for 10,000 iterations (as [8]). These confidence intervals demonstrate the uncertainty in the index values inherited from the baseline in 1970 and propagated through the time series.

## Results

### Geographic representation within the living planet index

**Fig 2:**
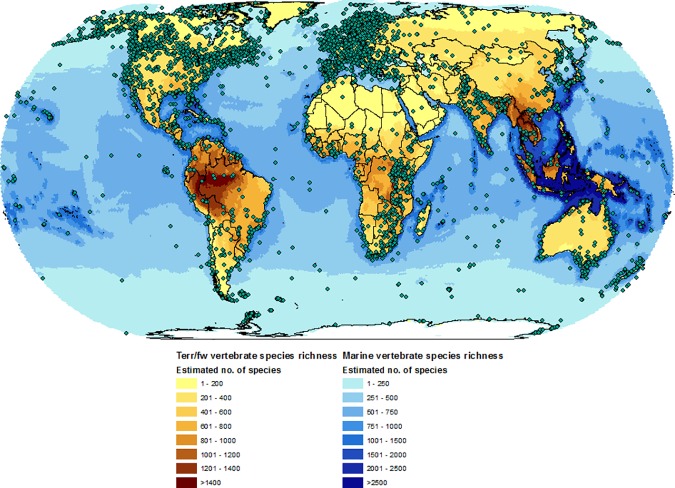
Global vertebrate richness map overlaid with populations recorded in the Living Planet Database. Species richness map reproduced from [20]

Global vertebrate richness overlaid with locations of populations currently recorded within the Living Planet Index shows biases towards temperate regions, which the Living Planet Index over-represents, and under-representation of tropical regions (Fig 2). Our comparison to a study on geographic bias in terrestrial ecological sites revealed that 63% of the terrestrial sites in the LPD occur in a protected area which is the same proportion as found in Martin et al. (*χ*^*2*^ = 0.004, *df* = 1, *p* = 0.95), and more than the expected 13% (*χ*^*2*^ = 883.83, *df* = 1, *p* = 0.00). For all woodland biomes, the LPI differs significantly to Martin et al.’s observed values except for Tundra (S2 Table). Compared to the expected number of sites across biomes, the LPI over-represents Tropical deciduous woodland and under-represents Tropical evergreen woodland (S3 Table). For values derived from an equal distribution of sites by global area, all other biomes except Tundra are over-represented while results are less clear by an assumed equal distribution among biomes (S3 Table). The pattern of representation in wealthy countries was similar to Martin et al. but overall results were mixed with over- und under-representation of high and low income countries compared to the number of sites expected (S4 Table). While comprising significantly more terrestrial sites from High income countries and significantly fewer sites from Upper middle income countries, representation is even when combining categories into higher (High and Upper middle) and lower (Lower middle and Low) groupings (S5 Table).

### Taxonomic representation and bias within the living planet index

**Fig 3:**
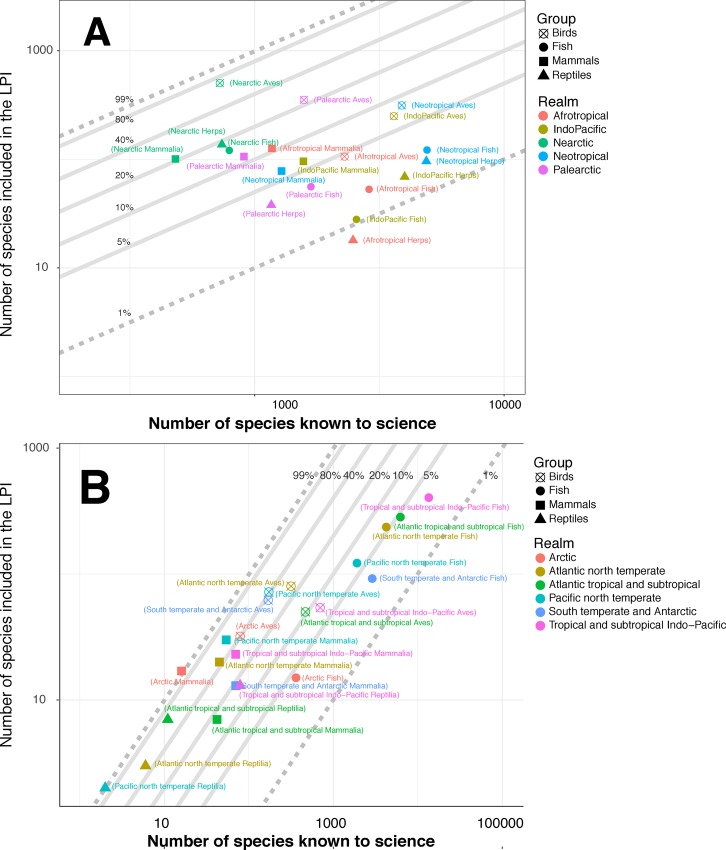
Comparison of number of known species and number of species recorded within the Living Planet Database. Colours represent different biogeographic realms, shapes indicate species groups and overlaid lines show 1 and 99% representation (dotted) and increments in between (solid). A–terrestrial and freshwater species and realms; B–marine species and realms

Fig 3 shows the geographic and taxonomic representation of species in the LPI. This representation is varied with 12 subsets representing between 1 and 10% and 7 subsets representing over 10% of known species in the terrestrial and freshwater systems (S6A Table). For the marine system, 6 subsets represent between 1 and 10% and 16 subsets represent 10% or more of known species (S6B Table). Afrotropical amphibians and reptiles (‘Afrotropical Herps’) represent less than 1% of known species and South temperate and Antarctic reptiles are currently not represented at all in the LPI database (0%, of a possible 3 species; not shown in figure). In the marine system, the highest representation of species is for Pacific north temperate reptiles (100%, 2 species). The highest terrestrial and freshwater representation is for Nearctic birds (68%, 492 species out of a possible 725 species) and the lowest is for Afrotropical reptiles and amphibians (0.7%, 18 species of a possible 2,480 species).

When compared to the expected diversity of species across realms, the significant results for birds and mammals show over-representation within terrestrial and freshwater realms with the exception of Afrotropical birds which are under-represented (Binomial test of proportions, see S7 Table). The taxonomic groups that are significantly under-represented in each terrestrial and freshwater realm are amphibians and reptiles, as well as fishes, the exception being Nearctic species which are all over-represented. For marine realms, the significant results for birds, mammals and reptiles show they are over-represented in all realms with the exception of South temperate and Antarctic reptiles where there is no representation of the three species (S8 Table). Fishes are a significantly under-represented group in the tropical and south temperate marine realms but are significantly over-represented in the Pacific north temperate.

### Impact of diversity weighting at the level of a realm: the palearctic

**Fig 4:**
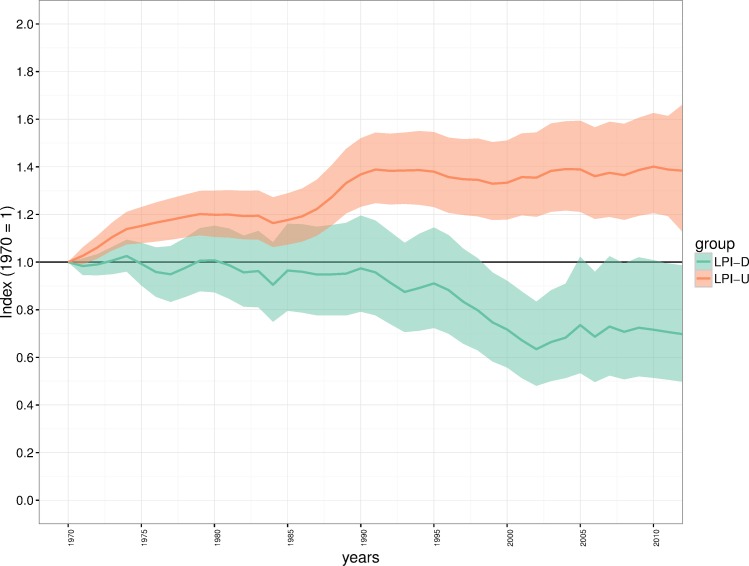
Comparison of the unweighted and diversity weighted Living Planet Index for the Palearctic realm. Green shows the unweighted index (LPI-U), orange shows the diversity weighted index (LPI-D). Solid coloured lines show the average trend and shaded regions show the 95% confidence interval of that trend.

Using the unweighted method (LPI-U) the index for the Palearctic realm shows an overall significant increase of 38.4% (95% CI: 12.7–66.2) over the period 1970–2012 (Fig 4). Using the diversity weighted method (LPI-D), the index for the Palearctic realm shows an overall significant decline of 30.3% (95% CI: -1.4 –-50.2). The LPI-D index for the Palearctic realm shows wider confidence intervals than the LPI-U index as well as a more undulating trend. When an unweighted average is used to calculate the Palearctic index, the group which contains the most species in the LPI database carries the most weight (S6A Table). The effect of using proportional weighting means that the influence of the over-represented groups such as birds and mammals has been reduced by over half and almost a fifth respectively, whereas the influence of fishes has been increased by over three-fold and amphibians/reptiles by over two-fold. This is compared to how much weight they would carry using the LPI-U approach where no taxonomic weighting is used.

### Applying the LPI-D approach to the global living planet index

**Fig 5:**
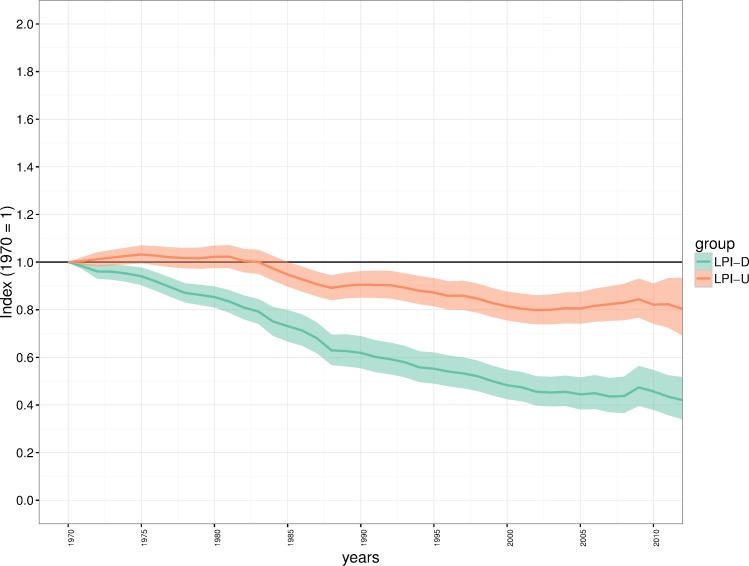
Comparison of the unweighted and diversity-weighted Living Planet Index for the global data set. Green shows the unweighted index (Global LPI-U), orange shows the diversity weighted index (Global LPI-D). Solid coloured lines show the average trend and shaded regions show the 95% confidence interval of that trend.

The global index produced using the LPI-D approach shows a decline of 58% (95% CI: -48.3 –-66.0) between 1970 and 2012 (Fig 5) which equates to an average annual decline of 2% per year. This result shows a greater rate of decline than the index calculated using the LPI-U approach which has an average annual decline of 0.52% per year and an overall decline of 19.7% (95% CI: -6.6 –-30.9), over the 42-year period. The confidence intervals around the LPI-U index are slightly wider than the LPI-D index illustrating greater uncertainty in the trend since 1970.

### System trends: terrestrial, freshwater and marine

**Fig 6:**
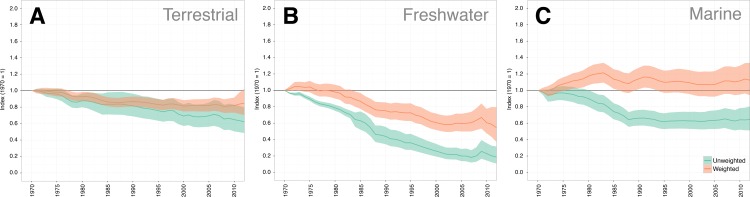
Comparison of the unweighted and diversity weighted Living Planet Index for each System (A -Terrestrial, B -Freshwater and C -Marine). In each case, green shows the unweighted index (LPI-U), orange shows the diversity weighted index (LPI-D). Solid coloured lines show the average trend and shaded regions show the 95% confidence interval of that trend.

The results of the LPI-D approach on the three system indices reveal that each show a greater decline than the LPI-U approach (Fig 6). The terrestrial index shows a 37.9% decline (95% CI: -20.4 –-51.5) from 1970 to 2012, averaging at a 1.13% decline per year. The marine index shows a similar decline of 35.6% (95% CI: -19.5 –-48.8) over the same period, with an average annual decline of 1.04% per year. The freshwater index shows a decline of greater magnitude, 81.5% (95% CI: -68.5 –-89.3) over the 42-year period and an average annual decline of 3.94% per year. Table 1 compares the weighted and unweighted indices for each system.

**Table 1:** Comparing the results of the weighted (LPI-D) and unweighted (LPI-U) indices in 2012. Confidence intervals are calculated from 10,000 bootstraps.

	**LPI-D index value in 2012**	**95% Confidence interval**	**LPI-U index value in 2012**	**95% Confidence interval**
**Terrestrial**	0.621	0.485–0.796	0.848	0.702–1.02
**Freshwater**	0.185	0.107–0.315	0.544	0.371–0.795
**Marine**	0.644	0.513–0.805	1.125	0.940–1.336

### The impact of low-representation groups

**Fig 7:**
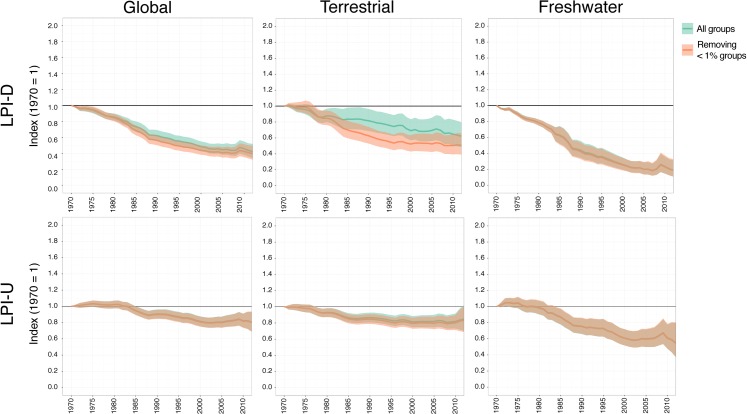
The impact of removing species groups for which the Living Planet Database has < 1% representation. Green trends show the Living Planet Index for all groups, orange trends show trends without less represented groups. Upper row shows trends calculated using the weighted (LPI-D) method, lower rows show the unweighted (LPI-U) method. Solid lines show the average trend, shaded regions show 95% confidence intervals. Stars (*) indicate when the final 2012 index values are significantly different.

To gauge the impact of less represented species groups on the indices, we explored the effect of removing them. If there was little impact, we would expect the average trend for the other groups that remain in the index to look similar after removal. Fig 7 compares the impact of removing these groups on global and system level trends using both the weighted and unweighted method. As no groups within the marine realm have < 1% representation, we only present the differences in global, freshwater and terrestrial indices. In general, the diversity weighted approach does not have a significant impact on the effect of removing these groups. In both weighted and unweighted cases for each index, no significant difference is seen when groups with less than 1% representation are removed. Each index shows a greater decline when these groups are removed, which is most noticeable in the Terrestrial LPI-D index but it is not significantly different. The exception is the Freshwater LPI-U index where there is a very marginal increase in the trend.

### Representation of threatened species

Comparing the proportion of species from each IUCN Red List category in the Living Planet Database with all assessed species on the IUCN Red List revealed some significant results for both threatened (CR, EN, VU) and non-threatened (NT/LR, LC) categories (Table 2). We find that Critically Endangered reptiles are significantly over-represented, along with Least Concern birds and amphibians, and Near Threatened/Lower Risk reptiles and fishes. The significantly under-represented groups are Near Threatened/Lower Risk birds, Least Concern reptiles and fishes, Endangered amphibians and fishes, and Vulnerable birds and amphibians. None of the categories for mammals showed significant over- or under- representation.

**Table 2:** Comparing the proportion of species within the Living Planet Database (LPI) and the IUCN Red List of Threatened Species (IUCN) for each Red List category (LC–Least Concern, NT/LR–Near Threatened/Lower Risk, VU—Vulnerable, EN–Endangered, CR–Critically Endangered).

Taxon	Category	LPI	IUCN	X^2^	Representation
**Mammalia**	CR	0.05	0.04	0.26	over
	EN	0.12	0.10	1.34	over
	VU	0.11	0.11	0.11	under
	NT/LR	0.07	0.07	0.19	under
	LC	0.64	0.66	0.44	under
** **	*Total # sp*.	*531*	*4753*	* *	
**Aves**	CR	0.02	0.02	0.21	over
	EN	0.04	0.04	0.17	under
	**VU**	**0.05**	**0.07**	10.34**	**under**
	**NT/LR**	**0.06**	**0.09**	12.75***	**under**
	**LC**	**0.82**	**0.76**	27.31***	**over**
** **	*Total # sp*.	*1415*	*10363*		
**Reptilia**	**CR**	**0.12**	**0.05**	15.72***	**over**
	EN	0.11	0.09	0.34	over
	VU	0.13	0.10	1.87	over
	**NT/LR**	**0.13**	**0.08**	4.04*	**over**
	**LC**	**0.49**	**0.68**	21.96***	**under**
** **	*Total # sp*.	*149*	*4244*		
**Amphibia**	CR	0.07	0.11	2.79	under
	**EN**	**0.06**	**0.17**	15.48***	**under**
	**VU**	**0.04**	**0.14**	12.96***	**under**
	NT/LR	0.08	0.08	0.00	under
	**LC**	**0.72**	**0.50**	35.12***	**over**
** **	*Total # sp*.	*178*	*4958*		
**Fishes**	CR	0.03	0.04	0.20	under
	**EN**	**0.03**	**0.05**	4.22*	**under**
	VU	0.09	0.10	0.96	under
	**NT/LR**	**0.07**	**0.05**	5.65*	**over**
	**LC**	**0.63**	**0.75**	45.45***	**under**
** **	*Total # sp*.	*602*	*12093*		

Chi-squared values are given for the binomial test of proportions, with significance levels indicated.

*p < 0.05.

∗∗p < 0.01.

∗∗∗p < 0.001.

presentation indicates whether the given group is ‘over’ or ‘under’ represented. Mammals, birds and amphibians have been comprehensively assessed by the IUCN.

When we subsetted the threatened species to include only those that have been assessed under Criterion A (a reduction in population size), we found more significance in the results between the proportions in the LPI and the IUCN Red List (S9 Table). All three threat categories are significantly over-represented for mammals, reptiles and fishes. Critically endangered and Endangered birds are significantly over-represented whereas Vulnerable birds are significantly under-represented. There were no significant results for amphibians.

## Discussion

Trends in abundance of species populations are a crucial indicator of biodiversity [28,29] and can provide early warnings of declines prior to species qualifying for high levels of extinction risk [30]. Consequently, this metric has been recommended as an Essential Biodiversity Variable [31], and, its use in geometric mean abundance indicators such as the Living Planet Index (LPI), is part of the mechanism to monitor biodiversity and assess progress towards the Aichi Targets.

The Living Planet Database (LPD), which underpins the LPI, relies on the collation of data from available sources such as government reports, scientific articles and research programmes which represents a cost effective method to develop a global biodiversity indicator. However, it necessarily suffers from a variety of publication biases arising for reasons such as lack of resources or infrastructure for monitoring, logistical difficulties in accessing sites or barriers to the dissemination of data into the public realm [12]. This is exacerbated by a tendency for monitoring to occur in areas where scientists live and work [21,32]. Across many of the species groups that are surveyed within the LPD, we see both significant over- and under- representation in comparison to the estimated number of species (S7 Table, S8 Table, Fig 3). The data tend to be over-represented for temperate bird and mammal species, and under-represented for most species groups in tropical realms and for marine fishes. We also find a geographic bias in the terrestrial data portion of the LPD towards protected areas, tropical deciduous woodland and some wealthy countries, at the same time as under-representation of tropical evergreen woodland biomes.

While the geographic and taxonomic bias we demonstrate in the LPI is consistent with other studies [8,33] and comparable data sets [21], the spatial mismatch between the known diversity of vertebrate species and the available data (Fig 2) could lead to inaccurate estimates of status and trends in biodiversity. More specifically, trends that equally weight these species groups (as in the ‘traditional’ Living Planet Index) will be significantly biased by the disproportionate representation of these groups, skewing the calculation of trends in global wildlife abundance. Given the need for developed indicators of biodiversity and the overriding challenges of obtaining globally comprehensive biodiversity data [12], we have outlined an approach to deal with bias as an interim solution in lieu of attaining more representative monitoring data. This weighted approach (LPI-D) suggests that, on average, species populations within the Palearctic may have declined by 30.3% as opposed to increasing in abundance by 38.4% (Fig 4) in the unweighted index (LPI-U). The difference is also notable at the global level where the LPI-U suggests a decline of 19.7%, compared to a significantly larger declines of 58% in the LPI-D.

Declines appear to be masked in the LPI-U as a result of a high proportion of well monitored, increasing populations in temperate regions in the data set. Weighting by species diversity in the LPI-D thus distributes the responsibility for the index across regions and taxa according to species richness. However, tropical regions tend to have higher richness and a greater proportion of threatened species [34], so this method may introduce another bias by placing a high proportion of weight on groups that may be less well monitored, under-represented, or more likely to be categorized as threatened. Comparing the proportion of threatened species within the LPI database to the IUCN Red List, we find that Critically Endangered reptiles are the only threatened group which is over-represented, while Endangered and Vulnerable amphibians are under-represented (Table 2). Conversely, we see significant results for nearly all groups when we examine only those threatened species from the analysis that have been assessed using Criterion A (S9 Table).

The implication of this is complex to interpret. As threatened species assessed under Criterion A are significantly over-represented in all groups except for amphibians, we can infer that the LPI has a bias towards negative population trends. However the impact may be partially tempered by the proportional weighting at taxonomic group level. For example, amphibians, which are not significantly over-represented by threatened species, along with reptiles, are given the highest weighting among the terrestrial species and the second highest weighting among freshwater species. Furthermore, species threatened under other criteria may be experiencing population declines but sufficient data are just not available to contribute to the Red Listing assessment. What is also important to note is that the majority of fish species (745 out of 1,369 species) have not yet been assessed by the IUCN Red List and a further 40 species are assessed as Data Deficient so these species were not included in this analysis.

Accounting for the diversity of species using the LPI-D method allows the LPI to be calculated in a more taxonomically representative way. However, it would clearly be more beneficial to continue to improve species representation within the LPD. The rate with which new data are incorporated is relatively constant (S1 Fig), as a wealth of data remains available in the literature. Manual entry of these data is a critical limitation in growing biodiversity databases such as the LPD, so tools for automating this process would be of value, e.g. working relationships and support with scientific journals to identify useful research papers and the data they contain [35]. New technologies such as remote sensing may also provide ways to improve the spatial coverage of data [36], and incorporating other data types such as occurrence or opportunistic data (e.g. from citizen science [37]) may help expand taxonomic coverage as abundance data is rare for non-vertebrates. Encouragingly, improvements will happen as existing biodiversity databases continue to be augmented and techniques to harness the power of citizen science projects improve [38]. In addition, initiatives to harmonise and standardise existing biodiversity databases are underway to enhance the current resource base for monitoring global biodiversity [39]. The demand for measures to report on biodiversity change however remains a challenge [40] and one where improving our resource base will not provide answers fast enough.

As well as addressing taxonomic disparity in the data set, the LPI-D approach accounts for the broad scale geographic bias present in the LPD by placing more weight on the largely tropical, more species-rich realms. However, issues of coverage still remain at smaller spatial scales which this approach does not tackle. For example, the data from the Palearctic realm is largely from Europe and there is much less coverage in Asia (Fig 2). Likewise in the Afrotropics, eastern and southern Africa are better represented than western and central Africa. For the marine system, data tend to be clustered near the coasts which is where most known impact from human activity occurs [41] but also the areas of higher species richness [42]. Understanding whether and how these patterns bias the trends in the LPI will be an important continuation of this work and one which is hard to untangle given the inferred impact of different types of bias. For example, the bias towards data from protected areas might suggest the LPI would show a greater decline if counterfactuals from unprotected sites were equally monitored, on the assumption that protection has a positive effect on population trends. Improving the coverage of Data Deficient species, as categorised by the IUCN Red List, might introduce negative trends if these species are likely to be threatened, as has been predicted for terrestrial mammals [43]. Alternatively, declines may be exacerbated by a prevalence of coastal marine data; areas of high human impact and where many heavily exploited commercial fish stocks are monitored.

We note that weighting by species diversity is only one of a number of potential weightings that could be applied to make the trends more ‘representative’. Other approaches have been used, for example, to account for the differing proportion of a species’ total population across different countries [15]. Depending on the question of interest, other methods of weighting could also be explored such as weighting by genetic diversity, functional diversity, biomes or other metrics. As well as the use we have outlined for the global scale, the application of weighting by species diversity could be applied when developing a national biodiversity indicator when species lists are readily available for the country in question. As the Convention on Biological Diversity requires Parties to report on their biodiversity trends, having a method that can be adapted at smaller scales is essential.

A limitation of our current approach is that it is reliant on reasonable species lists, which are known to change over time and may be of lower quality for less studied groups and regions. Estimates for the number of as yet unidentified birds and mammals are small (e.g. ~10–15 species), but the number of unidentified amphibians, reptiles and fish are much larger with respective estimates of 57%, 13% and 22% undescribed [44]. These latter groups would therefore be given even greater weight, suggesting that vertebrate populations may be declining, on average, even more rapidly that we currently estimate. As estimates of the known number of species improve, the relative weighting of species groups can be updated to better estimate overall trends.

Our analysis suggests that prior estimates of the trends in global wildlife populations may have underestimated their global decline. This appears to be due to those well monitored groups for which we have disproportionate amounts of data (predominantly in the Nearctic and Palearctic) declining less than those species in more speciose regions for which we have proportionally less data. We might expect that as the weighted index places more weight on less monitored groups in more species-rich regions, we would be exaggerating the declines in abundance–as we might expect these groups to be declining more. For example we know that tropical vertebrate populations are in worse decline than those in temperate regions [45] and that amphibians are threatened with a greater risk of extinction than mammals or birds [46]. However, we note that when we remove those species groups for which we have very little data (< 1% species), the overall trends decline more (Fig 7), potentially suggesting that overall declines may be worse than we currently present. We urgently need more data for these groups to better determine their trends.

## Supporting Information

S1 Appendix Assessing geographic bias in the LPD. (DOCX)

S1 Fig The cumulative number of population time series in the global LPI from 2006 to 2016. (DOCX)

S2 Fig The boundaries for land and marine realms used for the geographical divisions of the LPI database. Terrestrial realm data from Olson et al., (2001) and marine realms were drawn in ArcGIS 10.2.2 for Desktop.
(DOCX)

S1 Table Mapping of terrestrial biomes in the LPD to those in Martin *et al*. Asterisks denote significant differences in Martin *et al*.
(DOCX)

S2 Table Test of proportions for unique locations in the LPI compared to observed values in Martin *et al* (2012) with significance levels indicated (*p < 0.05, ∗∗p < 0.01, ∗∗∗p < 0.001). (DOCX)

S3 Table Test of proportions for unique locations in the LPI compared to expected values by area and distribution in Martin *et al* (2012) with significance levels indicated (*p < 0.05, ∗∗p < 0.01, ∗∗∗p < 0.001). Asterisks denote significant differences in Martin *et al*.
(DOCX)

S4 Table Test of proportions for unique locations by country in the LPI compared to expected values in Martin *et al* (2012) with significance levels indicated (*p < 0.05, ∗∗p < 0.01, ∗∗∗p < 0.001). Asterisks denote significant differences in Martin *et al*.
(DOCX)

S5 Table Test of proportions for unique locations by income category in the LPI compared to expected values in Martin *et al* (2012) with significance levels indicated (*p < 0.05, ∗∗p < 0.01, ∗∗∗p < 0.001). (DOCX)

S6 Table Known vertebrate species (‘Global estimate’) for A. terrestrial and freshwater system and B. marine system, compared to species recorded within the LPI database, and the proportion that this represents of the global estimate.
(DOCX)

S7 Table Comparing the proportion of terrestrial and freshwater species within the Living Planet Database (LPI) and the estimated known number of species (Known species) for each biogeographic realm and class. Chi-squared values are given for the binomial test of proportions, with significance levels indicated (*p < 0.05, **p < 0.01, ***p < 0.001). ‘Representation’ indicates whether the given group is ‘over’ or ‘under’ represented.
(DOCX)

S8 Table Comparing the proportion marine species within the Living Planet Database (LPI) and the estimated known number of species (Known species) for each biogeographic realm and class. Chi-squared values are given for the binomial test of proportions, with significance levels indicated (*p < 0.05, **p < 0.01, ***p < 0.001). ‘Representation’ indicates whether the given group is ‘over’ or ‘under’ represented.
(DOCX)

S9 Table Comparing the proportion of species within the Living Planet Database (LPI) and the IUCN Red List of Threatened Species (IUCN) for each Red List category (LC–Least Concern, NT/LR–Near Threatened/Lower Risk, VU—Vulnerable, EN–Endangered, CR–Critically Endangered). Only threatened species listed under Criterion A were included. Chi-squared values are given for the binomial test of proportions, with significance levels indicated (*p < 0.05, **p < 0.01, ***p < 0.001). Representation indicates whether the given group is ‘over’ or ‘under’ represented. Mammals, birds and amphibians have been comprehensively assessed by the IUCN.
(DOCX)

S10 Table Terrestrial and freshwater weightings applied to taxa/realm subsets within the global LPI. The values also represent the weighting applied to the data when calculating the system LPIs.
(DOCX)

S11 Table Marine weightings applied to taxa/realm subsets within the global LPI. The values also represent the weighting applied to the data for when calculating the system LPIs.
(DOCX)

S12 Table Terrestrial and freshwater realm weightings applied to data. (DOCX)

S13 Table Marine realm weightings applied to data. (DOCX)

## References

[1] Collen B, Nicholson E (2014) Taking the measure of change. Science 346: 166–167. 10.1126/science.1255772 25278506

[2] Dirzo R, Young HS, Galetti M, Ceballos G, Isaac NJB, et al (2014) Defaunation in the Anthropocene. Science 345: 401–406. 10.1126/science.1251817 25061202

[3] Tittensor DP, Walpole M, Hill SLL, Boyce DG, Britten GL, et al (2014) A mid-term analysis of progress toward international biodiversity targets. Science 346: 241–244. 10.1126/science.1257484 25278504

[4] De Vos JM, Joppa LN, Gittleman JL, Stephens PR, Pimm SL (2015) Estimating the normal background rate of species extinction. Conservation Biology 29: 452–462. 10.1111/cobi.12380 25159086

[5] Ceballos G, Ehrlich PR, Barnosky AD, García A, Pringle RM, et al (2015) Accelerated modern human–induced species losses: Entering the sixth mass extinction. Science Advances 1. 10.1126/sciadv.1400253 PMC4640606 26601195

[6] SCBD (2010) COP-10 Decision X/2. In: Secretariat of the convention on biological diversity, editor.

[7] WWF (2016) Living Planet Report 2016: Risk and resilience in a new era WWF International, Gland, Switzerland.

[8] Collen B, Loh J, Whitmee S, McRae L, Amin R, et al (2009) Monitoring Change in Vertebrate Abundance: the Living Planet Index. Conservation Biology 23: 317–327. 10.1111/j.1523-1739.2008.01117.x 19040654

[9] Loh J, Green RE, Ricketts T, Lamoreux J, Jenkins M, et al (2005) The Living Planet Index: using species population time series to track trends in biodiversity. Philosophical Transactions of the Royal Society of London B: Biological Sciences 360: 289–295. 10.1098/rstb.2004.1584 15814346 PMC1569448

[10] Boakes EH, McGowan PJK, Fuller RA, Chang-qing D, Clark NE, et al (2010) Distorted Views of Biodiversity: Spatial and Temporal Bias in Species Occurrence Data. PLoS Biol 8: e1000385 10.1371/journal.pbio.1000385 20532234 PMC2879389

[11] Yesson C, Brewer PW, Sutton T, Caithness N, Pahwa JS, et al (2007) How Global Is the Global Biodiversity Information Facility? PLoS ONE 2: e1124 10.1371/journal.pone.0001124 17987112 PMC2043490

[12] Collen B, Ram M, Zamin T, McRae L (2008) The tropical biodiversity data gap: addressing disparity in global monitoring. Tropical Conservation Science 1: 75–88.

[13] Pereira HM, Navarro LM, Martins IS (2012) Global Biodiversity Change: The Bad, the Good, and the Unknown. Annual Review of Environment and Resources 37: 25–50.

[14] Nicholson E, Collen B, Barausse A, Blanchard JL, Costelloe BT, et al (2012) Making Robust Policy Decisions Using Global Biodiversity Indicators. PLoS ONE 7: e41128 10.1371/journal.pone.0041128 22815938 PMC3399804

[15] Gregory RD, van Strien A, Vorisek P, Gmelig Meyling AW, Noble DG, et al (2005) Developing indicators for European birds. Philosophical Transactions of the Royal Society of London B: Biological Sciences 360: 269–288. 10.1098/rstb.2004.1602 15814345 PMC1569455

[16] van Swaay CAM, Nowicki P, Settele J, van Strien AJ (2008) Butterfly monitoring in Europe: methods, applications and perspectives. Biodiversity and Conservation 17: 3455–3469.

[17] Jones JPG, Collen B, Atkinson G, Baxter PWJ, Bubb P, et al (2011) The Why, What, and How of Global Biodiversity Indicators Beyond the 2010 Target. Conservation Biology 25: 450–457. 10.1111/j.1523-1739.2010.01605.x 21083762

[18] Loh J, Randers J, MacGillivray A, Kapos V, Groombridge B, et al. (1998) Living Planet Report 1998. WWF, Gland, Switzerland.

[19] UN (2015) Transforming our world: the 2030 Agenda for Sustainable Development. A/RES/70/1. https://sustainabledevelopment.un.org/post2015/transformingourworld.

[20] Baillie J, Griffiths J, Turvey S, Loh J, Collen B (2010) Evolution Lost: status & trends of the world's vertebrates Zoological Society of London, United Kingdom.

[21] Martin LJ, Blossey B, Ellis E (2012) Mapping where ecologists work: biases in the global distribution of terrestrial ecological observations. Frontiers in Ecology and the Environment 10: 195–201.

[22] World Wildlife Fund (2006) WildFinder: Online database of species distributions, ver. Jan-06. www.worldwildlife.org/WildFinder.

[23] Abell R, Thieme ML, Revenga C, Bryer M, Kottelat M, et al (2008) Freshwater Ecoregions of the World: A New Map of Biogeographic Units for Freshwater Biodiversity Conservation. BioScience 58: 403–414.

[24] IUCN (2016) The IUCN Red List of Threatened Species. Version 2016–2. http://www.iucnredlist.org/. Downloaded on 22nd November 2016.

[25] Uetz PJHe (2014) The Reptile Database, http://www.reptile-database.org, accessed April 15, 2014.

[26] OBIS (2014) Distribution records of marine vertebrate species (numerous data sets). Ocean Biogeographic Information System Intergovernmental Oceanographic Commission of UNESCO. http://www.iobis.org. Accessed: 2014-10-31.

[27] McRae L, Deinet S, Freeman R (2016) Data from: The diversity-weighted Living Planet Index: controlling for taxonomic bias in a global biodiversity indicator. Dryad Digital Repository. 10.1371/journal.pone.0169156 PMC5207715 28045977

[28] Santini L, Belmaker J, Costello MJ, Pereira HM, Rossberg AG, et al Assessing the suitability of diversity metrics to detect biodiversity change. Biological Conservation.

[29] Buckland ST, Studeny AC, Magurran AE, Illian JB, Newson SE (2011) The geometric mean of relative abundance indices: a biodiversity measure with a difference. Ecosphere 2: 1–15.

[30] Collen B, McRae L, Deinet S, De Palma A, Carranza T, et al (2011) Predicting how populations decline to extinction. Philosophical Transactions of the Royal Society B: Biological Sciences 366: 2577–2586. 21807738 10.1098/rstb.2011.0015 PMC3138608

[31] Pereira HM, Ferrier S, Walters M, Geller GN, Jongman RHG, et al (2013) Essential Biodiversity Variables. Science 339: 277–278. 10.1126/science.1229931 23329036

[32] Pyšek P, Richardson DM, Pergl J, Jarošík V, Sixtová Z, et al Geographical and taxonomic biases in invasion ecology. Trends in Ecology & Evolution 23: 237–244. 10.1016/j.tree.2008.02.002 18367291

[33] Proença V, Martin LJ, Pereira HM, Fernandez M, McRae L, et al Global biodiversity monitoring: From data sources to Essential Biodiversity Variables. Biological Conservation.

[34] Grenyer R, Orme CDL, Jackson SF, Thomas GH, Davies RG, et al (2006) Global distribution and conservation of rare and threatened vertebrates. Nature 444: 93–96. 10.1038/nature05237 17080090

[35] Huang X, Qiao G (2011) Biodiversity databases should gain support from journals. Trends in Ecology & Evolution 26: 377–378. 21665319 10.1016/j.tree.2011.05.006

[36] Pettorelli N, Wegmann M, Skidmore A, Mücher S, Dawson TP, et al (2016) Framing the concept of satellite remote sensing essential biodiversity variables: challenges and future directions. Remote Sensing in Ecology and Conservation: n/a-n/a.

[37] Isaac NJB, van Strien AJ, August TA, de Zeeuw MP, Roy DB (2014) Statistics for citizen science: extracting signals of change from noisy ecological data. Methods in Ecology and Evolution 5: 1052–1060.

[38] Pimm SL, Jenkins CN, Abell R, Brooks TM, Gittleman JL, et al (2014) The biodiversity of species and their rates of extinction, distribution, and protection. Science 344. 10.1126/science.1246752 24876501

[39] Kissling WD, Hardisty A, García EA, Santamaria M, De Leo F, et al (2015) Towards global interoperability for supporting biodiversity research on essential biodiversity variables (EBVs). Biodiversity 16: 99–107.

[40] Walpole M, Almond REA, Besançon C, Butchart SHM, Campbell-Lendrum D, et al (2009) Tracking Progress Toward the 2010 Biodiversity Target and Beyond. Science 325: 1503–1504. 10.1126/science.1175466 19762630

[41] Halpern BS, Walbridge S, Selkoe KA, Kappel CV, Micheli F, et al (2008) A Global Map of Human Impact on Marine Ecosystems. Science 319: 948–952. 10.1126/science.1149345 18276889

[42] Tittensor DP, Mora C, Jetz W, Lotze HK, Ricard D, et al (2010) Global patterns and predictors of marine biodiversity across taxa. Nature 466: 1098–1101. 10.1038/nature09329 20668450

[43] Bland LM, Collen B, Orme CDL, Bielby J (2015) Predicting the conservation status of data-deficient species. Conservation Biology 29: 250–259. 10.1111/cobi.12372 25124400

[44] Scheffers BR, Joppa LN, Pimm SL, Laurance WF (2012) What we know and don’t know about Earth's missing biodiversity. Trends in Ecology & Evolution 27: 501–510. 22784409 10.1016/j.tree.2012.05.008

[45] McRae L, Freeman R, Deinet S (2014) 'The Living Planet Index' in: Living Planet Report 2014: species and spaces, people and places WWF, Gland, Switzerland.

[46] Stuart SN, Chanson JS, Cox NA, Young BE, Rodrigues ASL, et al (2004) Status and Trends of Amphibian Declines and Extinctions Worldwide. Science 306: 1783–1786. 10.1126/science.1103538 15486254

