# Supplementary material for: The Diversity-Weighted Living Planet Index: Controlling for Taxonomic Bias in a Global Biodiversity Indicator
Source: PLoS One. 2017 Jan 3;12(1):e0169156. doi: 10.1371/journal.pone.0169156 (PMC5207715; doi:10.1371/journal.pone.0169156)
Supplement: S1 Appendix — (DOCX) [file pone.0169156.s001.docx]

# S1 Appendix. Assessing geographic bias in the LPD

***Method***

We compared our data with Martin *et al.* (2012) who describe several geographic biases including the overrepresentation of PAs, temperate woodlands and wealthy countries in study sites from recent publications. To use a comparable data set, we selected only terrestrial populations from the LPD and unique sites. We also only included those sites that have a specific location recorded – this avoids the use of sites which are a mid-point of a large survey area.

Protected areas – the populations in the LPD are already coded as to whether they occur in a protected area. We looked at the proportion of sites that are in protected areas as denoted in the LPD assessed using World Database on Protected Areas (IUCN and UNEP-WCMC, 2016).

Biomes – Martin *et al*. used Ramankutty & Foley’s Potential Natural Vegetation Cover (Ramankutty and Foley, 1999) to categorise biomes. In the LPD, the biomes have been categorised using WWF Ecoregions (Olson et al, 2001). We matched up the categories (Table S1) focussing only on woodland biomes as these were the ones highlighted in Martin *et al*. We compared the proportion of sites in each biome to the observed and expected proportions in Martin *et al*.

Wealthy countries – we used the categorisation from Martin *et al.* to look at the proportion of sites in wealthy and other countries, and combined for different country income categories as defined by the World Bank (World Bank, 2012).

***References***

# Martin LJ, Blossey B, Ellis E. (2012) Mapping where ecologists work: biases in the global distribution of terrestrial ecological observations. Frontiers in Ecology and the Environment 10(4):195-201.

# IUCN and UNEP-WCMC (2016), The World Database on Protected Areas (WDPA) [On-line], June 2016, Cambridge, UK: UNEP-WCMC. Available at: [www.protectedplanet.net](http://www.protectedplanet.net/).

# Ramankutty, N., and J.A. Foley (1999). Estimating historical changes in global land cover: Crop lands from 1700 to 1992. Global Biogeochemical Cycles 13(4), 997-1027.

# Olson DM, Dinerstein E, Wikramanayake ED, Burgess ND, Powell GVN, Underwood EC, et al. (2001) Terrestrial Ecoregions of the World: A New Map of Life on Earth: A new global map of terrestrial ecoregions provides an innovative tool for conserving biodiversity. BioScience 51(11):933-8.

# World Bank (2012) World Bank list of economies (July 2012).
