# Supplementary figures and images for: The Diversity-Weighted Living Planet Index: Controlling for Taxonomic Bias in a Global Biodiversity Indicator
Source: PLoS One. 2017 Jan 3;12(1):e0169156. doi: 10.1371/journal.pone.0169156 (PMC5207715; doi:10.1371/journal.pone.0169156)

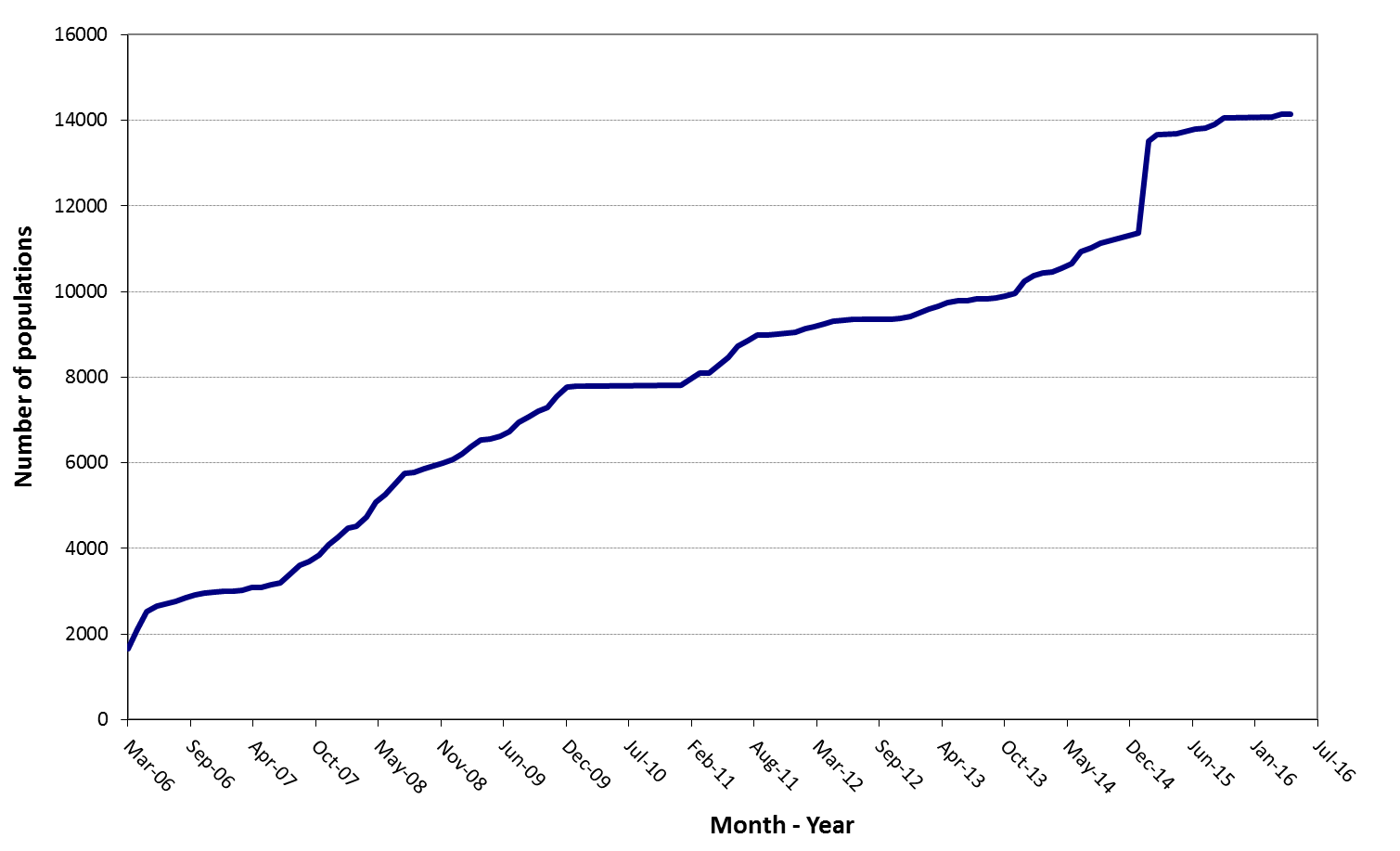


S1 Fig. The cumulative number of population time series in the global LPI from 2006 to 2016.

Supplement: S1 Fig — (DOCX) [file pone.0169156.s002.docx]
