# Supplementary material for: The Diversity-Weighted Living Planet Index: Controlling for Taxonomic Bias in a Global Biodiversity Indicator
Source: PLoS One. 2017 Jan 3;12(1):e0169156. doi: 10.1371/journal.pone.0169156 (PMC5207715; doi:10.1371/journal.pone.0169156)
Supplement: S2 Fig — Terrestrial realm data from Olson et al., (2001) and marine realms were drawn in ArcGIS 10.2.2 for Desktop. (DOCX) [file pone.0169156.s003.docx]

S2 Fig. The boundaries for land and marine realms used for the geographical divisions of the LPI database. Terrestrial realm data from Olson et al., (2001) and marine realms were drawn in ArcGIS 10.2.2 for Desktop.
