# Supplementary material for: The Diversity-Weighted Living Planet Index: Controlling for Taxonomic Bias in a Global Biodiversity Indicator
Source: PLoS One. 2017 Jan 3;12(1):e0169156. doi: 10.1371/journal.pone.0169156 (PMC5207715; doi:10.1371/journal.pone.0169156)
Supplement: S1 Table — Asterisks denote significant differences in Martin et al. (DOCX) [file pone.0169156.s004.docx]

| **Biome (Martin)** | **Biome (LPI)** |
| --- | --- |
| Tropical evergreen woodland* | Tropical & subtropical coniferous forests |
| Tropical deciduous woodland* | Tropical dry broadleaf forests / Tropical moist broadleaf forests |
| Temperate evergreen woodland* | Temperate coniferous forests |
| Temperate deciduous woodland* / Mixed woodland* | Temperate broadleaf and mixed forests |
| Boreal woodland | Boreal forests & taiga |
| Tundra* | Tundra |
