# Supplementary material for: The Diversity-Weighted Living Planet Index: Controlling for Taxonomic Bias in a Global Biodiversity Indicator
Source: PLoS One. 2017 Jan 3;12(1):e0169156. doi: 10.1371/journal.pone.0169156 (PMC5207715; doi:10.1371/journal.pone.0169156)
Supplement: S2 Table — (DOCX) [file pone.0169156.s005.docx]

| **Biome (Martin)** | **Representation (Martin)** | **Proportion (LPI)** | **Proportion (Martin, observed)** | **χ2** |  |
| --- | --- | --- | --- | --- | --- |
| Tropical evergreen woodland | over | 0.01 | 0.14 | 129.36 | *** |
| Tropical deciduous woodland | under | 0.18 | 0.02 | 204.55 | *** |
| Temperate evergreen woodland | over | 0.07 | 0.11 | 8.53 | ** |
| Temperate deciduous woodland / Mixed woodland | over | 0.20 | 0.31 | 30.42 | *** |
| Boreal woodland | NS | 0.09 | 0.08 | 0.20 | NS |
| Tundra | under | 0.07 | 0.03 | 15.17 | *** |
