# Supplementary material for: The Diversity-Weighted Living Planet Index: Controlling for Taxonomic Bias in a Global Biodiversity Indicator
Source: PLoS One. 2017 Jan 3;12(1):e0169156. doi: 10.1371/journal.pone.0169156 (PMC5207715; doi:10.1371/journal.pone.0169156)
Supplement: S3 Table — Asterisks denote significant differences in Martin et al. (DOCX) [file pone.0169156.s006.docx]

|  |  | **By area (Martin, expected)** | | | | **By equal distribution (Martin, expected)** | | | |
| --- | --- | --- | --- | --- | --- | --- | --- | --- | --- |
| **Biome (Martin)** | **Proportion (LPI)** | **Proportion** | **χ2** |  | **Representation** | **Proportion** | **χ2** |  | **Representation** |
| Tropical evergreen woodland* | 0.01 | 0.13 | 114.81 | *** | under | 0.08 | 63.60 | *** | under |
| Tropical deciduous woodland* | 0.18 | 0.05 | 116.86 | *** | over | 0.08 | 49.76 | *** | over |
| Temperate evergreen woodland* | 0.07 | 0.04 | 13.93 | *** | over | 0.08 | 1.04 | NS | NS |
| Temperate deciduous woodland / Mixed woodland* | 0.20 | 0.15 | 11.32 | *** | over | 0.17 | 5.59 | NS | NS |
| Boreal woodland | 0.09 | 0.06 | 4.81 | * | over | 0.08 | 0.04 | NS | NS |
| Tundra* | 0.07 | 0.05 | 1.84 | NS | NS | 0.08 | 1.46 | NS | NS |
