# Supplementary material for: The Diversity-Weighted Living Planet Index: Controlling for Taxonomic Bias in a Global Biodiversity Indicator
Source: PLoS One. 2017 Jan 3;12(1):e0169156. doi: 10.1371/journal.pone.0169156 (PMC5207715; doi:10.1371/journal.pone.0169156)
Supplement: S4 Table — Asterisks denote significant differences in Martin et al. (DOCX) [file pone.0169156.s007.docx]

| **Country** | **Income** | **Proportion  (LPI)** | **Proportion (Martin, expected)** | **χ2** | **Sig** | **Representation** |
| --- | --- | --- | --- | --- | --- | --- |
| Afghanistan | Low | 0.00 | 0.00 | 3.16 | NS | NS |
| Albania | Lower middle | 0.00 | 0.00 | 0.00 | NS | NS |
| Algeria | Upper middle | 0.00 | 0.02 | 15.56 | *** | under |
| American Samoa | Upper middle | 0.00 | 0.00 | NA | NA | NA |
| Andorra | High | 0.00 | 0.00 | NA | NA | NA |
| Angola | Upper middle | 0.00 | 0.01 | 5.50 | * | under |
| Antigua & Barbuda | Upper middle | 0.00 | 0.00 | 3.87 | * | over |
| Argentina | Upper middle | 0.02 | 0.02 | 0.64 | NS | NS |
| Armenia | Lower middle | 0.00 | 0.00 | 0.00 | NS | NS |
| Aruba | High | 0.00 | 0.00 | NA | NA | NA |
| Australia | High | 0.03 | 0.06 | 12.31 | *** | under |
| Austria | High | 0.00 | 0.00 | 0.00 | NS | NS |
| Azerbaijan | Upper middle | 0.00 | 0.00 | 0.00 | NS | NS |
| Bahrain | High | 0.00 | 0.00 | 0.00 | NS | NS |
| Bangladesh | Low | 0.00 | 0.00 | 0.03 | NS | NS |
| Barbados | High | 0.00 | 0.00 | NA | NA | NA |
| Belarus | Upper middle | 0.00 | 0.00 | 0.00 | NS | NS |
| Belgium | High | 0.00 | 0.00 | 0.00 | NS | NS |
| Belize | Lower middle | 0.00 | 0.00 | 0.00 | NS | NS |
| Benin | Low | 0.00 | 0.00 | 0.00 | NS | NS |
| Bhutan | Lower middle | 0.00 | 0.00 | 0.00 | NS | NS |
| Bolivia | Lower middle | 0.00 | 0.01 | 6.41 | * | under |
| Bosnia & Herzegovina | Upper middle | 0.00 | 0.00 | 0.00 | NS | NS |
| Botswana | Upper middle | 0.00 | 0.00 | 2.76 | NS | NS |
| Brazil | Upper middle | 0.02 | 0.07 | 29.33 | *** | under |
| Brunei Darussalam | High | 0.00 | 0.00 | 0.00 | NS | NS |
| Bulgaria | Upper middle | 0.00 | 0.00 | 0.00 | NS | NS |
| Burkina Faso | Low | 0.00 | 0.00 | 0.70 | NS | NS |
| Burundi | Low | 0.00 | 0.00 | 0.00 | NS | NS |
| Cambodia | Low | 0.00 | 0.00 | 0.00 | NS | NS |
| Cameroon | Lower middle | 0.00 | 0.00 | 0.00 | NS | NS |
| Canada | High | 0.16 | 0.07 | 49.59 | *** | over |
| Central African Republic | Low | 0.01 | 0.00 | 0.05 | NS | NS |
| Chad | Low | 0.00 | 0.01 | 3.98 | * | under |
| Chile | Upper middle | 0.01 | 0.01 | 0.41 | NS | NS |
| China | Upper middle | 0.01 | 0.07 | 52.53 | *** | under |
| Colombia | Upper middle | 0.00 | 0.01 | 6.80 | ** | under |
| Comoros | Low | 0.00 | 0.00 | 0.04 | NS | NS |
| Congo | Lower middle | 0.00 | 0.00 | 1.13 | NS | NS |
| Congo, DRC | Low | 0.01 | 0.02 | 6.12 | * | under |
| Costa Rica* | Upper middle | 0.00 | 0.00 | 1.40 | NS | NS |
| Côte d'Ivoire | Lower middle | 0.01 | 0.00 | 0.60 | NS | NS |
| Croatia | High | 0.00 | 0.00 | 0.35 | NS | NS |
| Cuba | Upper middle | 0.00 | 0.00 | 0.00 | NS | NS |
| Cyprus | High | 0.00 | 0.00 | 0.00 | NS | NS |
| Czech Republic | High | 0.00 | 0.00 | 0.19 | NS | NS |
| Denmark | High | 0.00 | 0.00 | 2.83 | NS | NS |
| Djibouti | Lower middle | 0.00 | 0.00 | 0.00 | NS | NS |
| Dominica | Upper middle | 0.00 | 0.00 | 0.04 | NS | NS |
| Dominican Republic | Upper middle | 0.00 | 0.00 | 0.00 | NS | NS |
| Ecuador | Upper middle | 0.00 | 0.00 | 0.03 | NS | NS |
| Egypt | Lower middle | 0.00 | 0.01 | 5.77 | * | under |
| El Salvador | Lower middle | 0.00 | 0.00 | 0.00 | NS | NS |
| Equatorial Guinea | High | 0.00 | 0.00 | 0.00 | NS | NS |
| Eritrea | Low | 0.00 | 0.00 | 0.00 | NS | NS |
| Estonia | High | 0.00 | 0.00 | 0.00 | NS | NS |
| Ethiopia | Low | 0.01 | 0.01 | 0.00 | NS | NS |
| Falkland Islands | Not listed | 0.00 | 0.00 | 0.00 | NS | NS |
| Fiji | Lower middle | 0.00 | 0.00 | 0.00 | NS | NS |
| Finland | High | 0.02 | 0.00 | 17.69 | *** | over |
| France | High | 0.02 | 0.00 | 13.69 | *** | over |
| French Guiana | Not listed | 0.00 | 0.00 | 0.00 | NS | NS |
| Gabon | Upper middle | 0.00 | 0.00 | 0.61 | NS | NS |
| Georgia | Lower middle | 0.00 | 0.00 | 0.00 | NS | NS |
| Germany* | High | 0.02 | 0.00 | 12.28 | *** | over |
| Ghana | Lower middle | 0.01 | 0.00 | 4.33 | * | over |
| Greece | High | 0.00 | 0.00 | 0.00 | NS | NS |
| Greenland* | High | 0.00 | 0.00 | 1.06 | NS | NS |
| Grenada | Upper middle | 0.00 | 0.00 | NA | NA | NA |
| Guadeloupe | Not listed | 0.00 | 0.00 | 0.00 | NS | NS |
| Guatemala | Lower middle | 0.00 | 0.00 | 0.00 | NS | NS |
| Guinea | Low | 0.00 | 0.00 | 0.00 | NS | NS |
| Guinea-Bissau | Low | 0.00 | 0.00 | 0.00 | NS | NS |
| Guyana | Lower middle | 0.00 | 0.00 | 0.33 | NS | NS |
| Haiti | Low | 0.00 | 0.00 | 0.00 | NS | NS |
| Honduras | Lower middle | 0.00 | 0.00 | 0.00 | NS | NS |
| Hungary | High | 0.00 | 0.00 | 0.00 | NS | NS |
| Iceland | High | 0.00 | 0.00 | 0.00 | NS | NS |
| India | Lower middle | 0.05 | 0.02 | 12.09 | *** | over |
| Indonesia | Lower middle | 0.01 | 0.01 | 3.83 | Near | (under) |
| Iran | Upper middle | 0.00 | 0.01 | 3.45 | NS | NS |
| Iraq | Lower middle | 0.00 | 0.00 | 1.72 | NS | NS |
| Ireland | High | 0.00 | 0.00 | 2.27 | NS | NS |
| Isle of Man | High | 0.00 | 0.00 | 0.00 | NS | NS |
| Israel* | High | 0.00 | 0.00 | 0.00 | NS | NS |
| Italy | High | 0.01 | 0.00 | 9.49 | ** | over |
| Jamaica | Upper middle | 0.00 | 0.00 | 0.00 | NS | NS |
| Japan | High | 0.00 | 0.00 | 0.00 | NS | NS |
| Jersey | Not listed | 0.00 | 0.00 | NA | NA | NA |
| Jordan | Upper middle | 0.00 | 0.00 | 0.00 | NS | NS |
| Kazakhstan | Upper middle | 0.00 | 0.02 | 17.97 | *** | under |
| Kenya | Low | 0.02 | 0.00 | 13.22 | *** | over |
| Kuwait | High | 0.00 | 0.00 | 0.00 | NS | NS |
| Kyrgyzstan | Low | 0.00 | 0.00 | 0.25 | NS | NS |
| Laos | Lower middle | 0.00 | 0.00 | 0.45 | NS | NS |
| Latvia | Upper middle | 0.00 | 0.00 | 0.00 | NS | NS |
| Lebanon | Upper middle | 0.00 | 0.00 | 0.00 | NS | NS |
| Lesotho | Lower middle | 0.00 | 0.00 | 0.00 | NS | NS |
| Liberia | Low | 0.00 | 0.00 | 0.00 | NS | NS |
| Libya | Upper middle | 0.00 | 0.01 | 10.38 | ** | under |
| Liechtenstein | High | 0.00 | 0.00 | NA | NA | NA |
| Lithuania | Upper middle | 0.00 | 0.00 | 0.00 | NS | NS |
| Luxembourg | High | 0.00 | 0.00 | 0.00 | NS | NS |
| Macedonia | Upper middle | 0.00 | 0.00 | 0.00 | NS | NS |
| Madagascar | Low | 0.01 | 0.00 | 0.38 | NS | NS |
| Malawi | Low | 0.00 | 0.00 | 0.81 | NS | NS |
| Malaysia | Upper middle | 0.00 | 0.00 | 0.11 | NS | NS |
| Mali | Low | 0.00 | 0.01 | 5.55 | * | under |
| Malta | High | 0.00 | 0.00 | 0.05 | NS | NS |
| Martinique | Not listed | 0.00 | 0.00 | 0.00 | NS | NS |
| Mauritania | Low | 0.00 | 0.01 | 4.06 | * | under |
| Mauritius | Upper middle | 0.00 | 0.00 | 1.06 | NS | NS |
| Mayotte | Not listed | 0.00 | 0.00 | NA | NA | NA |
| Mexico | Upper middle | 0.01 | 0.02 | 0.00 | NS | NS |
| Moldova | Lower middle | 0.00 | 0.00 | 0.00 | NS | NS |
| Mongolia | Lower middle | 0.00 | 0.01 | 3.03 | NS | NS |
| Montenegro | Upper middle | 0.00 | 0.00 | 0.01 | NS | NS |
| Montserrat | Not listed | 0.00 | 0.00 | NA | NA | NA |
| Morocco | Lower middle | 0.01 | 0.00 | 0.24 | NS | NS |
| Mozambique | Low | 0.01 | 0.01 | 0.00 | NS | NS |
| Myanmar | Low | 0.00 | 0.01 | 3.37 | NS | NS |
| Namibia | Upper middle | 0.00 | 0.01 | 1.40 | NS | NS |
| Nepal | Low | 0.01 | 0.00 | 11.56 | *** | over |
| Netherlands | High | 0.00 | 0.00 | 0.00 | NS | NS |
| Netherlands Antilles | Not listed | 0.00 | 0.00 | NA | NA | NA |
| New Caledonia | High | 0.00 | 0.00 | 0.00 | NS | NS |
| New Zealand | High | 0.01 | 0.00 | 7.47 | ** | over |
| Nicaragua | Lower middle | 0.00 | 0.00 | 0.00 | NS | NS |
| Niger | Low | 0.00 | 0.01 | 7.13 | ** | under |
| Nigeria | Lower middle | 0.00 | 0.01 | 0.93 | NS | NS |
| North Korea | Low | 0.00 | 0.00 | 0.01 | NS | NS |
| Norway | High | 0.02 | 0.00 | 13.40 | *** | over |
| Oman | High | 0.00 | 0.00 | 0.00 | NS | NS |
| Pakistan | Lower middle | 0.05 | 0.01 | 42.84 | *** | over |
| Panama* | Upper middle | 0.01 | 0.00 | 6.26 | * | over |
| Papua New Guinea | Lower middle | 0.00 | 0.00 | 1.97 | NS | NS |
| Paraguay | Lower middle | 0.00 | 0.00 | 1.50 | NS | NS |
| Peru | Upper middle | 0.00 | 0.01 | 4.08 | * | under |
| Philippines | Lower middle | 0.00 | 0.00 | 0.83 | NS | NS |
| Poland | High | 0.01 | 0.00 | 2.25 | NS | NS |
| Portugal | High | 0.00 | 0.00 | 0.89 | NS | NS |
| Puerto Rico* | High | 0.01 | 0.00 | 14.20 | *** | over |
| Qatar | High | 0.00 | 0.00 | 0.00 | NS | NS |
| Reunion | Not listed | 0.00 | 0.00 | 0.00 | NS | NS |
| Romania | Upper middle | 0.00 | 0.00 | 0.05 | NS | NS |
| Russian Federation | Upper middle | 0.03 | 0.13 | 59.31 | *** | under |
| Rwanda | Low | 0.00 | 0.00 | 0.67 | NS | NS |
| Samoa | Lower middle | 0.00 | 0.00 | 0.00 | NS | NS |
| San Marino | High | 0.00 | 0.00 | NA | NA | NA |
| São Tomé & Principe | Lower middle | 0.00 | 0.00 | 0.00 | NS | NS |
| Saudi Arabia | High | 0.00 | 0.01 | 8.31 | ** | under |
| Senegal | Lower middle | 0.00 | 0.00 | 0.16 | NS | NS |
| Serbia | Upper middle | 0.00 | 0.00 | 0.00 | NS | NS |
| Sierra Leone | Low | 0.00 | 0.00 | 0.00 | NS | NS |
| Singapore | High | 0.00 | 0.00 | 0.00 | NS | NS |
| Slovakia | High | 0.00 | 0.00 | 1.44 | NS | NS |
| Slovenia | High | 0.00 | 0.00 | 0.00 | NS | NS |
| Solomon Islands | Lower middle | 0.00 | 0.00 | 0.00 | NS | NS |
| Somalia | Low | 0.00 | 0.00 | 3.18 | NS | NS |
| South Africa | Upper middle | 0.05 | 0.01 | 31.99 | *** | over |
| South Korea | High | 0.00 | 0.00 | 1.74 | NS | NS |
| Spain | High | 0.03 | 0.00 | 26.95 | *** | over |
| Sri Lanka | Lower middle | 0.00 | 0.00 | 0.00 | NS | NS |
| St Kitts & Nevis | High | 0.00 | 0.00 | 0.05 | NS | NS |
| St Lucia | Upper middle | 0.00 | 0.00 | 0.04 | NS | NS |
| St Pierre & Miquelon | Not listed | 0.00 | 0.00 | NA | NA | NA |
| St Vincent & the Grenadines | Upper middle | 0.00 | 0.00 | NA | NA | NA |
| Sudan | Lower middle | 0.00 | 0.02 | 16.84 | *** | under |
| Suriname | Upper middle | 0.00 | 0.00 | 0.05 | NS | NS |
| Swaziland | Lower middle | 0.00 | 0.00 | 0.77 | NS | NS |
| Sweden* | High | 0.02 | 0.00 | 11.11 | *** | over |
| Switzerland* | High | 0.01 | 0.00 | 11.64 | *** | over |
| Syria | Lower middle | 0.00 | 0.00 | 0.22 | NS | NS |
| Tajikistan | Low | 0.00 | 0.00 | 0.03 | NS | NS |
| Tanzania | Low | 0.03 | 0.01 | 24.04 | *** | over |
| Thailand | Upper middle | 0.00 | 0.00 | 0.00 | NS | NS |
| The Bahamas | High | 0.00 | 0.00 | 0.02 | NS | NS |
| The Gambia | Low | 0.00 | 0.00 | 0.00 | NS | NS |
| Timor-Leste | Lower middle | 0.00 | 0.00 | 0.00 | NS | NS |
| Togo | Low | 0.00 | 0.00 | 0.00 | NS | NS |
| Tonga | Lower middle | 0.00 | 0.00 | NA | NA | NA |
| Trinidad & Tobago | High | 0.00 | 0.00 | 0.00 | NS | NS |
| Tunisia | Upper middle | 0.00 | 0.00 | 0.09 | NS | NS |
| Turkey | Upper middle | 0.00 | 0.01 | 4.14 | * | under |
| Turkmenistan | Upper middle | 0.00 | 0.00 | 1.97 | NS | NS |
| Turks & Caicos Islands | High | 0.00 | 0.00 | NA | NA | NA |
| Uganda | Low | 0.02 | 0.00 | 24.60 | *** | over |
| Ukraine | Lower middle | 0.00 | 0.00 | 2.79 | NS | NS |
| United Arab Emirates | High | 0.00 | 0.00 | 0.00 | NS | NS |
| United Kingdom* | High | 0.02 | 0.00 | 20.66 | *** | over |
| United States* | High | 0.06 | 0.07 | 0.40 | NS | NS |
| Uruguay | Upper middle | 0.00 | 0.00 | 0.17 | NS | NS |
| Uzbekistan | Lower middle | 0.00 | 0.00 | 0.01 | NS | NS |
| Vanuatu | Lower middle | 0.00 | 0.00 | 0.00 | NS | NS |
| Venezuela | Upper middle | 0.00 | 0.01 | 1.85 | NS | NS |
| Vietnam | Lower middle | 0.01 | 0.00 | 0.57 | NS | NS |
| Virgin Islands (U.S.) | High | 0.00 | 0.00 | NA | NA | NA |
| Western Sahara | Not listed | 0.00 | 0.00 | 0.65 | NS | NS |
| Yemen | Lower middle | 0.00 | 0.00 | 1.89 | NS | NS |
| Zambia | Lower middle | 0.02 | 0.01 | 6.06 | * | over |
| Zimbabwe | Low | 0.01 | 0.00 | 5.40 | * | over |
