# Supplementary material for: The Diversity-Weighted Living Planet Index: Controlling for Taxonomic Bias in a Global Biodiversity Indicator
Source: PLoS One. 2017 Jan 3;12(1):e0169156. doi: 10.1371/journal.pone.0169156 (PMC5207715; doi:10.1371/journal.pone.0169156)
Supplement: S5 Table — (DOCX) [file pone.0169156.s008.docx]

| **Income category** | **No. of countries** | **Proportion (Martin, expected)** | **Proportion (LPI)** | **χ2** | **Sig** | **Representation** |
| --- | --- | --- | --- | --- | --- | --- |
| High | 57 | 0.25 | 0.49 | 137.21 | *** | over |
| Low | 36 | 0.13 | 0.15 | 2.42 | NS | NS |
| Lower middle | 47 | 0.16 | 0.17 | 0.37 | NS | NS |
| Not listed | 11 | 0.00 | 0.00 | 0.19 | NS | NS |
| Upper middle | 50 | 0.46 | 0.19 | 175.39 | *** | under |
| *Higher (High + Upper middle)* | *107* | *0.71* | *0.68* | *2.58* | *NS* | *NS* |
| *Lower (Low + Lower middle)* | *83* | *0.28* | *0.32* | *2.89* | *NS* | *NS* |
