# Supplementary material for: The Diversity-Weighted Living Planet Index: Controlling for Taxonomic Bias in a Global Biodiversity Indicator
Source: PLoS One. 2017 Jan 3;12(1):e0169156. doi: 10.1371/journal.pone.0169156 (PMC5207715; doi:10.1371/journal.pone.0169156)
Supplement: S6 Table — Known vertebrate species (‘Global estimate’) for A. terrestrial and freshwater system and B. marine system, compared to species recorded within the LPI database, and the proportion that this represents of the global estimate. (DOCX) [file pone.0169156.s009.docx]

| A |  | Species numbers | |  |  |
| --- | --- | --- | --- | --- | --- |
|  |  | Global estimate | LPI database | Proportion | |
| Amphibia and Reptilia | Afrotropical | 2480 | 18 | 0.01 | |
|  | IndoPacific | 3994 | 69 | 0.02 | |
|  | Nearctic | 739 | 137 | 0.19 | |
|  | Neotropical | 4879 | 96 | 0.02 | |
|  | Palearctic | 1166 | 42 | 0.04 | |
| Aves | Afrotropical | 2294 | 106 | 0.05 | |
|  | IndoPacific | 3616 | 249 | 0.07 | |
|  | Nearctic | 725 | 492 | 0.68 | |
|  | Neotropical | 3890 | 312 | 0.08 | |
|  | Palearctic | 1575 | 353 | 0.22 | |
| Mammalia | Afrotropical | 1173 | 126 | 0.11 | |
|  | IndoPacific | 1568 | 96 | 0.06 | |
|  | Nearctic | 481 | 101 | 0.21 | |
|  | Neotropical | 1282 | 78 | 0.06 | |
|  | Palearctic | 906 | 117 | 0.13 | |
| FW Fishes* | Afrotropical | - | 51 | 0.02 | |
|  | IndoPacific | - | 28 | 0.01 | |
|  | Nearctic | - | 121 | 0.15 | |
|  | Neotropical | - | 122 | 0.02 | |
|  | Palearctic | - | 56 | 0.03 | |

| B |  | Species numbers | |  |
| --- | --- | --- | --- | --- |
|  |  | Global estimate | LPI database | Proportion |
| Reptilia | Arctic | 0 | 0 | N/A |
|  | Atlantic north temperate | 6 | 3 | 0.50 |
|  | Atlantic tropical and subtropical | 11 | 7 | 0.64 |
|  | Pacific north temperate | 2 | 2 | 1.00 |
|  | South temperate and Antarctic | 3 | 0 | 0.00 |
|  | Tropical and subtropical Indo-Pacific | 79 | 13 | 0.16 |
| Aves | Arctic | 79 | 29 | 0.37 |
|  | Atlantic north temperate | 316 | 81 | 0.26 |
|  | Atlantic tropical and subtropical | 467 | 50 | 0.11 |
|  | Pacific north temperate | 172 | 61 | 0.35 |
|  | South temperate and Antarctic | 167 | 62 | 0.37 |
|  | Tropical and subtropical Indo-Pacific | 694 | 53 | 0.08 |
| Mammalia | Arctic | 16 | 16 | 1.00 |
|  | Atlantic north temperate | 45 | 20 | 0.44 |
|  | Atlantic tropical and subtropical | 42 | 6 | 0.14 |
|  | Pacific north temperate | 54 | 29 | 0.54 |
|  | South temperate and Antarctic | 70 | 13 | 0.19 |
|  | Tropical and subtropical Indo-Pacific | 70 | 20 | 0.29 |
| Fishes | Arctic | 291 | 15 | 0.05 |
|  | Atlantic north temperate | 1826 | 237 | 0.13 |
|  | Atlantic tropical and subtropical | 4454 | 280 | 0.06 |
|  | Pacific north temperate | 1681 | 121 | 0.07 |
|  | South temperate and Antarctic | 2721 | 91 | 0.03 |
|  | Tropical and subtropical Indo-Pacific | 11627 | 404 | 0.03 |

S6 Table. Known vertebrate species (‘Global estimate’) for A. terrestrial and freshwater systems and B. marine system, compared to species recorded within the LPI database, and the proportion that this represents of the global estimate. *The exact estimates for freshwater fishes based on Abell et al (2008) are not publicly available.
