# Supplementary material for: The Diversity-Weighted Living Planet Index: Controlling for Taxonomic Bias in a Global Biodiversity Indicator
Source: PLoS One. 2017 Jan 3;12(1):e0169156. doi: 10.1371/journal.pone.0169156 (PMC5207715; doi:10.1371/journal.pone.0169156)
Supplement: S7 Table — Chi-squared values are given for the binomial test of proportions, with significance levels indicated (*p < 0.05, **p < 0.01, ***p < 0.001). ‘Representation’ indicates whether the given group is ‘over’ or ‘under’ represented. (DOCX) [file pone.0169156.s010.docx]

| Realm | Taxon | LPI | Known species | X-squared | Significant? | Representation |
| --- | --- | --- | --- | --- | --- | --- |
| Afrotropical | Amphibia and Reptilia | 0.01 | 0.06 | 130.93 | *** | under |
| Afrotropical | Aves | 0.04 | 0.05 | 11.54 | *** | under |
| Afrotropical | Fishes | 0.02 | 0.07 | 101.09 | *** | under |
| Afrotropical | Mammalia | 0.05 | 0.03 | 30.75 | *** | over |
| IndoPacific | Amphibia and Reptilia | 0.02 | 0.09 | 147.55 | *** | under |
| IndoPacific | Aves | 0.09 | 0.08 | 1.09 |  | over |
| IndoPacific | Fishes | 0.01 | 0.06 | 118.02 | *** | under |
| IndoPacific | Mammalia | 0.04 | 0.04 | 2.32 |  | over |
| Nearctic | Amphibia and Reptilia | 0.05 | 0.02 | 142.94 | *** | over |
| Nearctic | Aves | 0.18 | 0.02 | 2595.10 | *** | over |
| Nearctic | Fishes | 0.04 | 0.02 | 84.12 | *** | over |
| Nearctic | Mammalia | 0.04 | 0.01 | 130.66 | *** | over |
| Neotropical | Amphibia and Reptilia | 0.03 | 0.11 | 165.70 | *** | under |
| Neotropical | Aves | 0.11 | 0.09 | 15.21 | *** | over |
| Neotropical | Fishes | 0.04 | 0.11 | 129.77 | *** | under |
| Neotropical | Mammalia | 0.03 | 0.03 | 0.19 |  | under |
| Palearctic | Amphibia and Reptilia | 0.02 | 0.03 | 13.96 | *** | under |
| Palearctic | Aves | 0.13 | 0.04 | 530.49 | *** | over |
| Palearctic | Fishes | 0.02 | 0.04 | 24.81 | *** | under |
| Palearctic | Mammalia | 0.04 | 0.02 | 52.55 | *** | over |

S7 Table. Comparing the proportion of terrestrial and freshwater species within the Living Planet Database (LPI) and the estimated known number of species (Known species) for each biogeographic realm and class. Chi-squared values are given for the binomial test of proportions, with significance levels indicated (*p < 0.05, ∗∗p < 0.01, ∗∗∗p < 0.001). ‘Representation’ indicates whether the given group is ‘over’ or ‘under’ represented.
