# Supplementary material for: The Diversity-Weighted Living Planet Index: Controlling for Taxonomic Bias in a Global Biodiversity Indicator
Source: PLoS One. 2017 Jan 3;12(1):e0169156. doi: 10.1371/journal.pone.0169156 (PMC5207715; doi:10.1371/journal.pone.0169156)
Supplement: S8 Table — Chi-squared values are given for the binomial test of proportions, with significance levels indicated (*p < 0.05, **p < 0.01, ***p < 0.001). ‘Representation’ indicates whether the given group is ‘over’ or ‘under’ represented. (DOCX) [file pone.0169156.s011.docx]

| Realm | Taxon | LPI | Known species | X-squared | Significant? | Representation |
| --- | --- | --- | --- | --- | --- | --- |
| Arctic | Aves | 0.02 | 0.00 | 107.94 | *** | over |
| Arctic | Fishes | 0.01 | 0.01 | 0.49 |  | under |
| Arctic | Mammalia | 0.01 | 0.00 | 130.87 | *** | over |
| Atlantic North Temperate | Aves | 0.05 | 0.01 | 205.11 | *** | over |
| Atlantic North Temperate | Fishes | 0.15 | 0.13 | 1.82 |  | over |
| Atlantic North Temperate | Mammalia | 0.01 | 0.00 | 88.50 | *** | over |
| Atlantic North Temperate | Reptilia | 0.00 | 0.00 | 10.16 | ** | over |
| Atlantic Tropical and Sub-tropical | Aves | 0.03 | 0.01 | 24.76 | *** | over |
| Atlantic Tropical and Sub-tropical | Fishes | 0.17 | 0.20 | 5.69 | * | under |
| Atlantic Tropical and Sub-tropical | Mammalia | 0.00 | 0.00 | 4.48 | * | over |
| Atlantic Tropical and Sub-tropical | Reptilia | 0.00 | 0.00 | 37.83 | *** | over |
| Pacific North Temperate | Aves | 0.04 | 0.01 | 223.25 | *** | over |
| Pacific North Temperate | Fishes | 0.08 | 0.06 | 5.18 | * | over |
| Pacific North Temperate | Mammalia | 0.02 | 0.00 | 155.48 | *** | over |
| Pacific North Temperate | Reptilia | 0.00 | 0.00 | 9.17 | ** | over |
| S.Temperate and Antarctic | Aves | 0.04 | 0.01 | 235.35 | *** | over |
| S.Temperate and Antarctic | Fishes | 0.06 | 0.09 | 23.18 | *** | under |
| S.Temperate and Antarctic | Mammalia | 0.01 | 0.00 | 18.57 | *** | over |
| S.Temperate and Antarctic | Reptilia | 0.00 | 0.00 | 0.00 |  | under |
| Tropical and Sub-tropical Indo-Pacific | Aves | 0.03 | 0.02 | 7.31 | ** | over |
| Tropical and Sub-tropical Indo-Pacific | Fishes | 0.25 | 0.43 | 201.20 | *** | under |
| Tropical and Sub-tropical Indo-Pacific | Mammalia | 0.01 | 0.00 | 54.76 | *** | over |
| Tropical and Sub-tropical Indo-Pacific | Reptilia | 0.01 | 0.00 | 15.06 | *** | over |

S8 Table. Comparing the proportion of marine species within the Living Planet Database (LPI) and the estimated known number of species (Known species) for each biogeographic realm and class. Chi-squared values are given for the binomial test of proportions, with significance levels indicated (*p < 0.05, ∗∗p < 0.01, ∗∗∗p < 0.001). ‘Representation’ indicates whether the given group is ‘over’ or ‘under’ represented.
