# Supplementary material for: The Diversity-Weighted Living Planet Index: Controlling for Taxonomic Bias in a Global Biodiversity Indicator
Source: PLoS One. 2017 Jan 3;12(1):e0169156. doi: 10.1371/journal.pone.0169156 (PMC5207715; doi:10.1371/journal.pone.0169156)
Supplement: S9 Table — Only threatened species listed under Criterion A were included. Chi-squared values are given for the binomial test of proportions, with significance levels indicated (*p < 0.05, **p < 0.01, ***p < 0.001). Representation indicates whether the given group is ‘over’ or ‘under’ represented. Mammals, birds and amphibians have been comprehensively assessed by the IUCN. (DOCX) [file pone.0169156.s012.docx]

| **Taxon** | **Category** | **LPI** | **IUCN** | **X^2^** | **Representation** |
| --- | --- | --- | --- | --- | --- |
| **Mammalia** | **CR** | **0.04** | **0.02** | **4.26*** | **over** |
|  | **EN** | **0.08** | **0.03** | **26.71***** | **over** |
|  | **VU** | **0.08** | **0.06** | **4.55*** | **over** |
|  | *Total # sp.* | *485* | *3985* |  |  |
| **Aves** | **CR** | **0.01** | **0.01** | **13.45***** | **over** |
|  | **EN** | **0.02** | **0.01** | **11.84***** | **over** |
|  | **VU** | **0.03** | **0.03** | **4.69E-29***** | **under** |
|  | *Total # sp.* | *1352* | *9438* |  |  |
| **Reptilia** | **CR** | **0.12** | **0.02** | **58.44***** | **over** |
|  | **EN** | **0.08** | **0.01** | **36.11***** | **over** |
|  | **VU** | **0.08** | **0.03** | **11.31***** | **over** |
|  | *Total # sp.* | *133* | *3458* |  |  |
| **Amphibia** | CR | 0.07 | 0.06 | 0.09 | over |
|  | **EN** | 0.03 | 0.02 | 0.97 | over |
|  | **VU** | 0.02 | 0.01 | 0.63 | over |
|  | *Total # sp.* | *170* | *3186* |  |  |
| **Fishes** | **CR** | **0.03** | **0.01** | **6.23*** | **over** |
|  | **EN** | **0.03** | **0.01** | **7.90**** | **over** |
|  | **VU** | **0.08** | **0.03** | **38.90***** | **over** |
|  | *Total # sp.* | *590* | *10381* |  |  |

**S9 Table. Comparing the proportion of species within the Living Planet Database (LPI) and the IUCN Red List of Threatened Species (IUCN) for each Red List category (LC – Least Concern, NT/LR – Near Threatened/Lower Risk, VU - Vulnerable, EN – Endangered, CR – Critically Endangered). Only threatened species listed under Criterion A were included. Chi-squared values are given for the binomial test of proportions, with significance levels indicated (*p < 0.05, ∗∗p < 0.01, ∗∗∗p < 0.001). Representation indicates whether the given group is ‘over’ or ‘under’ represented. Mammals, birds and amphibians have been comprehensively assessed by the IUCN.**
