# Supplementary material for: The Diversity-Weighted Living Planet Index: Controlling for Taxonomic Bias in a Global Biodiversity Indicator
Source: PLoS One. 2017 Jan 3;12(1):e0169156. doi: 10.1371/journal.pone.0169156 (PMC5207715; doi:10.1371/journal.pone.0169156)
Supplement: S10 Table — The values also represent the weighting applied to the data when calculating the system LPIs. (DOCX) [file pone.0169156.s013.docx]

|  |  | | Afrotropical | Nearctic | Neotropical | Palearctic | Indo-Pacific |
| --- | --- | --- | --- | --- | --- | --- | --- |
| Terrestrial groups | | Birds | 0.387 | 0.376 | 0.387 | 0.433 | 0.396 |
|  |  | Mammals | 0.197 | 0.249 | 0.127 | 0.249 | 0.172 |
|  |  | Reptiles and amphibians | 0.414 | 0.373 | 0.484 | 0.316 | 0.431 |
| Freshwater groups | | Fishes | 0.590 | 0.565 | 0.584 | 0.592 | 0.493 |
|  |  | Birds | 0.192 | 0.203 | 0.107 | 0.211 | 0.176 |
|  |  | Mammals | 0.009 | 0.013 | 0.010 | 0.015 | 0.008 |
|  |  | Reptiles and amphibians | 0.207 | 0.217 | 0.298 | 0.179 | 0.321 |

S10 Table. Terrestrial and freshwater weightings applied to taxa/realm subsets within the global LPI. The values also represent the weighting applied to the data when calculating the system LPIs.
