# Supplementary material for: The Diversity-Weighted Living Planet Index: Controlling for Taxonomic Bias in a Global Biodiversity Indicator
Source: PLoS One. 2017 Jan 3;12(1):e0169156. doi: 10.1371/journal.pone.0169156 (PMC5207715; doi:10.1371/journal.pone.0169156)
Supplement: S11 Table — The values also represent the weighting applied to the data for when calculating the system LPIs. (DOCX) [file pone.0169156.s014.docx]

|  | Arctic | Atlantic North Temperate | Atlantic Tropical and Sub-tropical | Pacific North Temperate | Tropical and Sub-tropical Indo-Pacific | South Temperate and Antarctic |
| --- | --- | --- | --- | --- | --- | --- |
| Reptiles | 0 | 0.001303 | 0.001630 | 0.000935 | 0.005505 | 0.000957 |
| Birds | 0.172867 | 0.068635 | 0.069353 | 0.080916 | 0.048714 | 0.054261 |
| Mammals | 0.035011 | 0.009774 | 0.006224 | 0.025257 | 0.004878 | 0.022342 |
| Fishes | 0.792123 | 0.920286 | 0.922791 | 0.892890 | 0.940901 | 0.922438 |

S11 Table. Marine weightings applied to taxa/realm subsets within the global LPI. The values also represent the weighting applied to the data for when calculating the system LPIs.
