# Supplementary material for: The Diversity-Weighted Living Planet Index: Controlling for Taxonomic Bias in a Global Biodiversity Indicator
Source: PLoS One. 2017 Jan 3;12(1):e0169156. doi: 10.1371/journal.pone.0169156 (PMC5207715; doi:10.1371/journal.pone.0169156)
Supplement: S12 Table — (DOCX) [file pone.0169156.s015.docx]

|  | Afrotropical | Nearctic | Neotropical | Palearctic | Indo-Pacific |
| --- | --- | --- | --- | --- | --- |
| Terrestrial LPI | 0.189738 | 0.061683 | 0.321132 | 0.116431 | 0.292168 |
| Freshwater LPI | 0.211701 | 0.060853 | 0.365550 | 0.123314 | 0.225576 |

S12 Table. Terrestrial and freshwater realm weightings applied to data.
