# Supplementary material for: The Diversity-Weighted Living Planet Index: Controlling for Taxonomic Bias in a Global Biodiversity Indicator
Source: PLoS One. 2017 Jan 3;12(1):e0169156. doi: 10.1371/journal.pone.0169156 (PMC5207715; doi:10.1371/journal.pone.0169156)
Supplement: S13 Table — (DOCX) [file pone.0169156.s016.docx]

|  | Arctic | Atlantic North Temperate | Atlantic Tropical and Sub-tropical | Pacific North Temperate | Tropical and Sub-tropical Indo Pacific | South Temperate and Antarctic |
| --- | --- | --- | --- | --- | --- | --- |
| Marine LPI | 0.014541 | 0.146489 | 0.214706 | 0.068026 | 0.456553 | 0.099685 |

S13 Table. Marine realm weightings applied to data.
